# Antifungal turbinmicin alters functional vesicle cargo

**DOI:** 10.1128/aac.00558-26

**Published:** 2026-06-22

**Authors:** Robert Zarnowski, Jeniel E. Nett, Tim S. Bugni, David Andes

**Affiliations:** 1 Department of Medicine, School of Medicine and Public Health, University of Wisconsin-Madison 5228, Madison, Wisconsin, USA; 2 School of Pharmacy, University of Wisconsin-Madison 5228, Madison, Wisconsin, USA; University Children's Hospital Münster, Münster, Germany

**Keywords:** turbinmicin, mechanism, biofilm, antifungal

## Abstract

*Candida* biofilms produce an extracellular matrix that sequesters antifungals, allowing cells to proliferate despite otherwise therapeutic concentrations. A prior study found turbinmicin to inhibit the release of the extracellular vesicles (EVs) required for matrix delivery and drug resistance. Here, we show that turbinmicin also alters the cargo loaded into EVs, decreasing the relative abundance of proteins that drive production of the drug-sequestering matrix. Turbinmicin displays a new dual antifungal action, disrupting both EV quantity and cargo.

## INTRODUCTION

Fungal biofilms withstand high concentrations of antifungals, up to 1,000-fold greater than those needed to eradicate non-biofilm, planktonic cells (1). The matrix functions to sequester antifungal drugs, reducing antifungal penetration to intracellular drug targets (2–4). Turbinmicin, a recently discovered natural product antifungal, exhibits broad-spectrum activity that notably includes efficacy against difficult-to-treat fungal biofilms (5, 6). Results of the initial investigation revealed a mechanism of action that involved inhibition of extracellular vesicle (EV) trafficking, a pathway critical for the production of an extracellular biofilm matrix. Docking studies suggested that turbinmicin targets Sec14p, a phospholipid transfer protein of the trans-Golgi export pathway for EVs (7). Turbinmicin treatment of biofilms formed by a variety of fungal pathogens was found to decrease the quantity of EVs produced, ultimately reducing the amount of protective matrix encasing the biofilm communities, and leading to antifungal susceptibility (8–12). However, it was unclear if the reduced EV quantity fully accounted for the profound susceptibility to antifungals that was observed.

As specific EV cargo components are critical for assembly of the extracellular matrix, we considered the possibility that turbinmicin may alter the cargo composition as a second mechanism of antibiofilm activity (9, 10). For *Candida* spp., the capacity of biofilms to withstand antifungals has been most closely linked to the presence of a mannan-glucan complex assembled in the matrix (3, 13–15). This matrix polysaccharide complex sequesters antifungals, resulting in a shielding phenomenon and the drug resistance phenotype observed for *Candida* biofilms. The cargo required for the production of this complex includes a variety of mannans and glucans and modification enzymes. The necessity of specific functional cargo constituents for this mechanism led us to question the impact of turbinmicin on the proteome of cargo loaded into vesicles.

**Fig 1:**
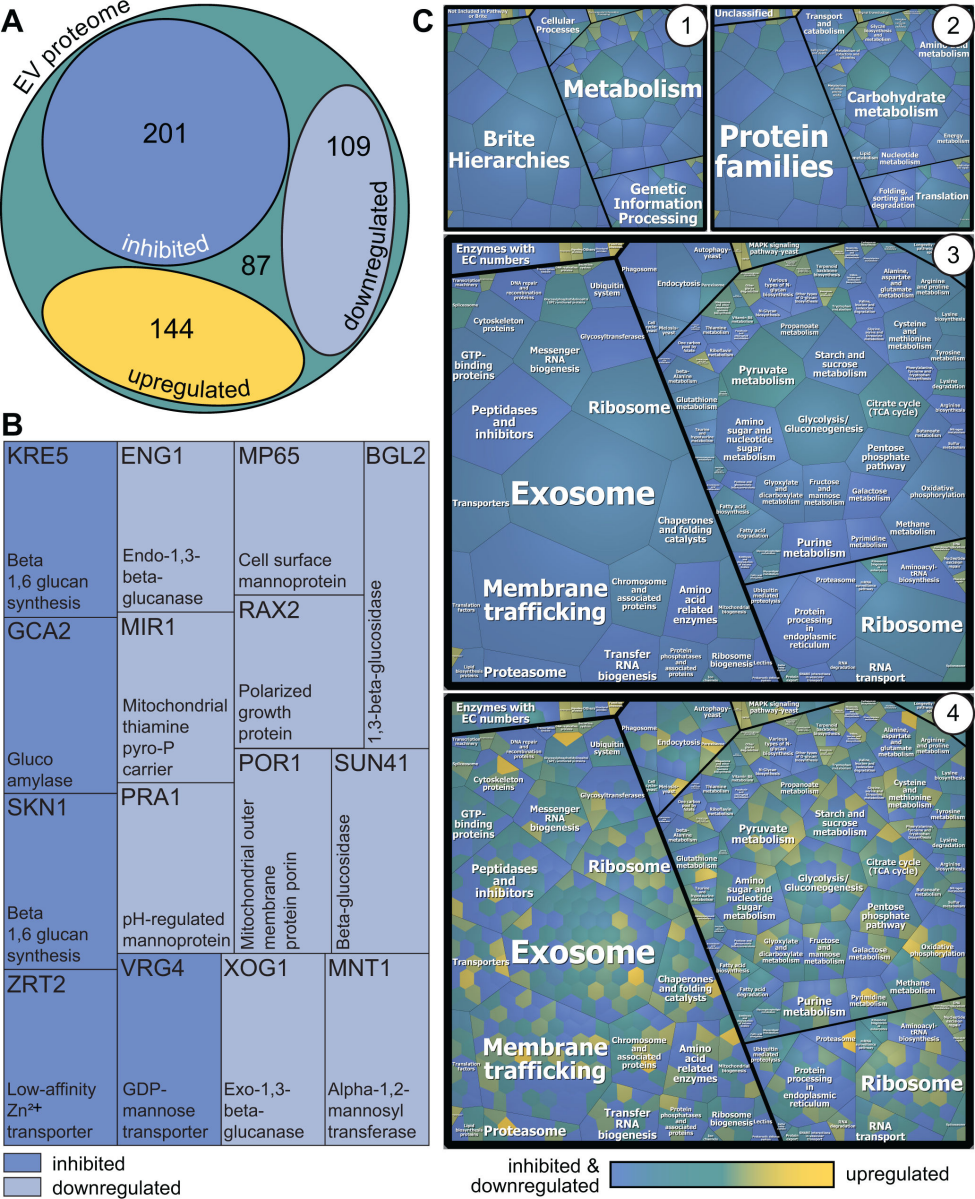
(**A**) Euler diagram depicting the qualitative profiling of *C. albicans* vesicle proteomes in wild-type and turbinmicin-treated biofilms. Inhibited proteins were absent. Downregulated proteins were reduced in abundance. Upregulated proteins were increased in abundance. Other proteins were present and unchanged in abundance. *N* = 3 pooled biofilm replicates. (**B**) Treemap of cargo constituent proteins absent or reduced in abundance with known function in mannan-glucan matrix sequestration of antifungals. (**C**) KEGG/BRITE-based functional mapping of *Candida* EV proteomes from turbinmicin-exposed biofilms. Clusters of inhibited (blue), downregulated (blue), unchanged (green), and upregulated (yellow) proteins are shown in a color gradient ramp spanning blue to yellow. Individual levels 4, 3, 2, and 1 represent identified proteins and are arranged inside higher-level regions according to their KEGG and BRITE functional category and pathway assignment using a Voronoi treemap layout.

To delineate the impact of turbinmicin on EV loading of specific cargo, we exposed mature *C. albicans* biofilms to subinhibitory concentrations of turbinmicin for 24 h and collected EVs for cargo proteomic analysis. Compared to EVs from untreated biofilms, we identified major changes in vesicle cargo upon turbinmicin treatment, with more than 50% of the untreated biofilm EV cargo proteins either absent or markedly reduced in abundance (decreased by >50%) in the treated biofilms (Fig. 1A). This finding shows that the impact of turbinmicin treatment extends beyond decreasing the quantity of EVs to additionally include alterations in the loaded EV cargo. We were particularly interested in the abundance of 15 cargo proteins previously demonstrated to produce and assemble the mannan-glucan complex. Strikingly, all the matrix mannan-glucan complex proteins were among the inhibited constituents (Fig. 1B) (3, 4, 10, 12, 13). We postulate this change in functional cargo loading is in part responsible for the anti-biofilm mechanism of turbinmicin.

Among the other protein cargo components that were altered in the drug-treated biofilm vesicles, we identified a wide range of putative functional categories including proteins implicated in biofilm formation and, not surprisingly, the vesicular pathway (Fig. 1C and Table S1). Of particular interest among the inhibited cargo is the finding of 20 mannan and glucan-modifying enzymes. It is possible that one or more of these vesicle cargo proteins may play a role in the construction of the mannan-glucan complex. How fungal pathogens regulate which proteins will be assembled into EV cargo is largely unknown. We speculate that the impact of turbinmicin on cargo composition is specific to its known mechanism of vesicle pathway inhibition. However, it is possible that a portion of the EV cargo changes may be due to drug-induced cell stress, as has been observed for drugs that do not target the vesicle pathway, such as caspofungin (16, 17).

Inhibition of the vesicle pathway is increasingly recognized as a valuable drug target in mammalian cells (18, 19). While drug discovery efforts have identified several compounds that inhibit vesicle production, only a few alter cargo. These drugs have been shown to be effective in treating a wide range of human diseases (19, 20). The present investigations provide evidence that supports dual anti-biofilm activities for turbinmicin, which result in both inhibition of EV production and an alteration of EV cargo.

## SUPPLEMENTAL MATERIAL

Supplemental material aac.00558-26-s0001.docx Supplemental methods; Table S1.

ASM does not own the copyrights to Supplemental Material that may be linked to, or accessed through, an article. The authors have granted ASM a non-exclusive, world-wide license to publish the Supplemental Material files. Please contact the corresponding author directly for reuse.
