# Supplementary material for: Antifungal turbinmicin alters functional vesicle cargo
Source: Antimicrob Agents Chemother. 2026 Jun 22;70(8):e00558-26. doi: 10.1128/aac.00558-26 (PMC13436242; doi:10.1128/aac.00558-26)
Supplement: Supplementary file 1 [file aac.00558-26-s0001.docx]

**Supplemental Information:**

**Methods**

Fungal strain, media and culture conditions: *C. albicans* SN250 was utilized for all studies. The fungal strain was stored in 15% glycerol frozen at −80 °C. Strains were routinely maintained on YPD agar plates (1% yeast extract, 2% Bacto™ peptone, 2% dextrose, 2% Bacto™ agar), whereas liquid cultures were grown in broth YPD (1% yeast extract, 2% Bacto™ peptone, 2% dextrose) rotating at 200 rpm at 30 °C. For biofilm assays, strains were cultured in filter sterilized Roswell Park Memorial Institute medium 1640 (RPMI), buffered with 4-morpholinepropanesulfonic acid (MOPS), and pH adjusted to 7.0. Biofilms were grown for 24 h after which one biofilm was exposed to turbinmicin at a concentration of 2 µg/mL for an additional 24 h. Control biofilm was grown for 48 h.

Biofilm vesicle isolation and characterization: Vesicles were prepared using a large-scale rolling bottle biofilm model system. The culture media was removed from the polystyrene bottles, filter sterilized, and concentrated down to 25 ml using a Vivaflow 200 unit (Sartorius AG) equipped with a Hydrosart 30 kDa cut-off membrane. In order to remove smaller cellular debris, the sample was centrifuged first at 10,000 × *g* for 1 h at 4 °C. The pellet was discarded, and the resulting supernatant was centrifuged again at 100,000 × *g* for 1.5 h at 4 °C. Next, the supernatant was discarded, and the pellet was then resuspended in 10 μl of PBS per bottle. The sample was filter sterilized at stored until further use at 4 °C. Accordingly to the standards for studies of EVs, the isolated EVs were further qualitatively validated by imaging flow cytometry, nanoparticle tracking analysis, and cryo-transmission electron microscopy (data not shown). To verify that the observed phenotypic effects in the EV addback assay occurred indeed due to the presence of EVs and was not driven by other co-purified non-vesicular contaminants, the cell-free EV fraction obtained after the ultracentrifugation step (100,000 × *g* for 1.5 h at 4 °C) was further processed using an exoEasy Maxi Kit (Qiagen). This kit uses generic membranes with high affinity for specific binding and recovery of EVs from cell culture supernatant. These EVs were then assessed in the EV addback assay which demonstrated comparable activity levels of both membrane-polished EVs and EVs prepared only by ultracentrifugation. Thus, we subsequently removed this step from our EV purification protocol in this study.

The mean particle size of the vesicle dispersions was determined using a Zetasizer Nano-ZS (Malvern Instruments). In order to obtain the optimal light scattering intensity, 10 μl of the vesicle suspension was added to 990 μl of PBS. All the measurements were carried out in triplicate at 25 °C. Vesicles were quantified using nanoparticle tracking analysis. EV samples were diluted in PBS to a final volume of 1 ml and pretested to obtain an ideal 30–100 particles per frame rate using a NanoSight NS300 system (Malvern). The following settings were applied: camera level was increased to 16 and camera gain to 2 until tested images were optimized and nanoparticles were distinctly visible without exceeding particle signal saturation. Each measurement consisted of five 1-min videos with a delay of 5 s between sample introduction and the start of the first measurement. For detection threshold analysis the counts were limited to 10–100 red crosses and no more than 5–7 blue crosses. Acquired data were analyzed using the NanoSight Software NTA 3.4 Build 3.4.003. At least 1000 events in total was tracked per sample in order to minimize data skewing based on single large particles[^40^](https://pmc.ncbi.nlm.nih.gov/articles/PMC8556236/#CR40).

Label-free gel-free proteomics: We had 2 groups: control vs treatment. For each group we grew 30 biofilms. We repeated this three times per group. Protein was isolated from each replicate from each group. Equal amounts of protein from each group were pooled yielding one aggregate sample group (treatment and control). Two hundred micrograms of proteins were extracted by precipitation with 15% Trichloroacetic acid (TCA)/60% acetone and then incubated at −20 °C for 30 min. The matrix or vesicle preparation was centrifuged at 16,000 × *g* for 10 min, and the resulting pellets were washed twice with ice-cold acetone, followed by an ice-cold MeOH wash. Pelleted proteins were resolubilized and denatured in 10 μl of 8 M urea in 100 mM NH_4_HCO_3_ for 10 min, then diluted to 60 μl for tryptic digestion with the following reagents: 3 μl of 25 mM DTT, 4.5 μl of acetonitrile, 36.2 μl of 25 mM NH_4_HCO_3_, 0.3 μl of 1 M Tris-HCl, and 6 μl of 100 ng/μl Trypsin Gold solution in 25 mM NH_4_HCO_3_ (Promega). Digestion was conducted in two stages, first overnight at 37 °C, then additional 4 μl of trypsin solution were added and the mixture was incubated at 42 °C for an additional 2 h. The reaction was terminated by acidification with 2.5% TFA to a final concentration of 0.3% and then centrifuged at 16,000 × *g* for 10 min. Trypsin-generated peptides were analyzed by nanoLC-MS/MS using the Agilent 1100 nanoflow system (Agilent) connected to a hybrid linear ion trap-orbitrap mass spectrometer (LTQ-Orbitrap, Thermo Fisher Scientific) equipped with a nanoelectrospray ion source. Capillary HPLC was performed using an in-house fabricated column with an integrated electrospray emitter, as described elsewhere[^29^](https://pmc.ncbi.nlm.nih.gov/articles/PMC8556236/#CR29). Sample loading and desalting were achieved using a trapping column in line with the autosampler (Zorbax 300SB-C18, 5 μm, 5 × 0.3 mm, Agilent). The LTQ-Orbitrap was set to acquire MS/MS spectra in a data-dependent mode as follows: MS survey scans from 300 to 2000 *m*/*z* were collected in profile mode with a resolving power of 100,000. MS/MS spectra were collected on the five most abundant signals in each survey scan. Dynamic exclusion was employed to increase the dynamic range and maximize peptide identifications. Raw MS/MS data were searched against a concatenated *C. albicans* amino acid sequence database using an in-house MASCOT search engine[^30^](https://pmc.ncbi.nlm.nih.gov/articles/PMC8556236/#CR30). Identified proteins were further annotated and filtered to 1.5% peptide and 0.1% protein false-discovery-rate with Scaffold Q + version 4.10.0 (Proteome Software Inc.) using the protein prophet algorithm[^31^](https://pmc.ncbi.nlm.nih.gov/articles/PMC8556236/#CR31). Proteomic data were mapped using Heatmapper[^32^](https://pmc.ncbi.nlm.nih.gov/articles/PMC8556236/#CR32). The mass spectrometry proteomics data have been deposited to the ProteomeXchange Consortium via the PRIDE [1] partner repository with the dataset identifier PXD076431 and 10.6019/PXD076431.

| Supplementary table 1. Proteomes of WT and turbinmicin-treated biofilms EVs. | | | | | | |
| --- | --- | --- | --- | --- | --- | --- |
| Gene locus | **UniProt ID** | **Gene name** | **Function** | **Control** | **Turbinmicin** | **Turbinmicin/ Control** |
| CAALFM_C202380WA | Q5ALL8 | POB3 | FACT complex subunit POB3 (Facilitates chromatin transcription complex subunit POB3) | 132855624 | 0 | Inhibited |
| CAALFM_C210150WA | A0A1D8PIN7 | U1766 | Uncharacterized protein | 8549529 | 0 | Inhibited |
| CAALFM_C201300CA | Q5AD72 | MNN24 | Alpha-1,2-mannosyltransferase MNN24 (EC 2.4.1.-) | 4537559 | 0 | Inhibited |
| CAALFM_C307470WA | A0A1D8PKU6 | EVP1 | Uncharacterized protein | 4094991 | 0 | Inhibited |
| CAALFM_C301830CA | P46592 | MNT2 | Glycolipid 2-alpha-mannosyltransferase 2 (EC 2.4.1.-) (Alpha-1,2-mannosyltransferase 2) | 4049937 | 0 | Inhibited |
| CAALFM_C301470WA | A0A1D8PJ64 | ARF3 | Arf3p | 3880165 | 0 | Inhibited |
| CAALFM_C208870CA | Q59SF7 | PIR1 | Cell wall mannoprotein PIR1 (Protein with internal repeats 1) | 3611024 | 0 | Inhibited |
| CAALFM_CR07150WA | Q59TZ8 | GLK1 | Phosphotransferase (EC 2.7.1.-) | 2515336 | 0 | Inhibited |
| CAALFM_CR10840CA | A0A1D8PUB4 | XYL2 | L-iditol 2-dehydrogenase | 2354716 | 0 | Inhibited |
| CAALFM_C110040WA | A0A1D8PER8 | ERO1 | ER oxidoreductin | 2070891 | 0 | Inhibited |
| CAALFM_C307180CA | Q5ADQ9 | BMT1 | Beta-mannosyltransferase 1 (EC 2.4.1.-) (WRY family protein 4) | 1771446 | 0 | Inhibited |
| CAALFM_C302510CA | Q5AEM6 | SAP8 | Candidapepsin-8 (EC 3.4.23.24) (ACP 8) (Aspartate protease 8) (Secreted aspartic protease 8) | 1667530 | 0 | Inhibited |
| CAALFM_C209740WA | Q59YF0 | SSO2 | Protein transport protein SSO2 | 1265529 | 0 | Inhibited |
| CAALFM_C301320CA | Q5AJ73 | SGA1 | 1,4-alpha-D-glucan glucohydrolase (EC 3.2.1.3) (Glucan 1,4-alpha-glucosidase) | 1264438 | 0 | Inhibited |
| CAALFM_C306120CA | Q8TGH6 | GDA1 | Guanosine-diphosphatase (GDPase) (EC 3.6.1.42) | 1258537 | 0 | Inhibited |
| CAALFM_C207900WA | A0A1D8PI00 | GDH2 | NAD-specific glutamate dehydrogenase (EC 1.4.1.2) | 1240459 | 0 | Inhibited |
| CAALFM_C305370CA | O42817 | RPS0 | 40S ribosomal protein S0 | 1218276 | 0 | Inhibited |
| CAALFM_C503280WA | A0A1D8PNN8 | CAM1-1 | Cam1-1p | 1159765 | 0 | Inhibited |
| CAALFM_C700940WA | Q5AFP8 | KEX1 | Pheromone-processing carboxypeptidase KEX1 (EC 3.4.16.6) (Carboxypeptidase D) | 1156065 | 0 | Inhibited |
| CAALFM_C104500WA | Q59RB8 | ICL1 | Isocitrate lyase | 957006 | 0 | Inhibited |
| CAALFM_C702570CA | P28870 | RBP1; RBP2 | FK506-binding protein 1 (FKBP) (EC 5.2.1.8) (Peptidyl-prolyl cis-trans isomerase) (PPIase) (Rapamycin-binding protein) | 937607 | 0 | Inhibited |
| CAALFM_CR07180WA | A0A1D8PTF1 | EMP46 | Emp46p | 887241 | 0 | Inhibited |
| CAALFM_C104290CA | Q8NJN3 | ACS2 | Acetyl-coenzyme A synthetase 2 (EC 6.2.1.1) (Acetate--CoA ligase 2) (Acyl-activating enzyme 2) | 853991 | 0 | Inhibited |
| CAALFM_C110300WA | Q5APQ8 | MNN12 | Putative alpha-1,3-mannosyltransferase MNN12 (EC 2.4.1.-) | 839644 | 0 | Inhibited |
| CAALFM_C202590WA | A0A1D8PGN5 | ZRT2 | Zinc transporter | 818293 | 0 | Inhibited |
| CAALFM_C504470CA | Q5AK88 | U3932 | RRM domain-containing protein | 791888 | 0 | Inhibited |
| CAALFM_C701650WA | A0A1D8PQW6 | U6559 | Transcription factor TFIIIC subunit | 790350 | 0 | Inhibited |
| CAALFM_CR04570CA | P39827 | CDC10 | Cell division control protein 10 | 782769 | 0 | Inhibited |
| CAALFM_C100710CA | A0A1D8PC97 | TUB2 | Tubulin beta chain | 762083 | 0 | Inhibited |
| CAALFM_CR02130WA | A0A1D8PS56 | ECM4 | Omega-class glutathione transferase | 759854 | 0 | Inhibited |
| CAALFM_C113160WA | Q5AL34 | PSA2 | Psa2p | 722916 | 0 | Inhibited |
| CAALFM_C108740CA | Q5AQ76 | SEC24 | Protein transport protein SEC24 | 722447 | 0 | Inhibited |
| CAALFM_C107040CA | Q5AB49 | U6200 | Probable pathogenesis-related protein CaO19.6200 | 681415 | 0 | Inhibited |
| CAALFM_C702810WA | Q5A5A0 | PRX1 | Thioredoxin peroxidase | 666805 | 0 | Inhibited |
| CAALFM_CR09160CA | A0A1D8PTW6 | ERG13 | 3-hydroxy-3-methylglutaryl coenzyme A synthase (HMG-CoA synthase) (EC 2.3.3.10) | 663846 | 0 | Inhibited |
| CAALFM_CR00510CA | A0A1D8PRQ1 | ADE1 | SAICAR synthetase (EC 6.3.2.6) | 625020 | 0 | Inhibited |
| CAALFM_C100180WA | Q5AB87 | RPL16A | Ribosomal 60S subunit protein L16A | 610654 | 0 | Inhibited |
| CAALFM_C102620CA | Q5AIA2 | HOM6 | Homoserine dehydrogenase (HDH) (EC 1.1.1.3) | 609620 | 0 | Inhibited |
| CAALFM_C601860CA | A0A1D8PPR5 | HCH1 | Hch1p | 603720 | 0 | Inhibited |
| CAALFM_C600840WA | Q59WW8 | GPX2 | Glutathione peroxidase | 584530 | 0 | Inhibited |
| CAALFM_CR01280CA | Q5A8A2 | NPC2 | Phosphatidylglycerol/phosphatidylinositol transfer protein (PG/PI-TP) | 550759 | 0 | Inhibited |
| CAALFM_C104890WA | A0A1D8PDE8 | YBN5 | Obg-like ATPase 1 | 543932 | 0 | Inhibited |
| CAALFM_C401510WA | Q5AML3 | U4633 | Oxidoreductase | 508597 | 0 | Inhibited |
| CAALFM_C208500WA | Q59Y21 | RSP5 | E3 ubiquitin-protein ligase (EC 2.3.2.26) | 503289 | 0 | Inhibited |
| CAALFM_C104830WA | A0A1D8PDE3 | SEC21 | Coatomer subunit gamma | 495762 | 0 | Inhibited |
| CAALFM_C500560WA | Q5A513 | U933 | E2 ubiquitin-conjugating protein | 489784 | 0 | Inhibited |
| CAALFM_C701710WA | A0A1D8PQW4 | U6553 | DUF3844 domain-containing protein | 474118 | 0 | Inhibited |
| CAALFM_C108980CA | A0A1D8PEG2 | ZWF1 | Glucose-6-phosphate dehydrogenase | 472031 | 0 | Inhibited |
| CAALFM_C603340CA | A0A1D8PQ59 | GLC3 | 1,4-alpha-glucan-branching enzyme (EC 2.4.1.18) (Glycogen-branching enzyme) | 464611 | 0 | Inhibited |
| CAALFM_C203600WA | Q5AHD6 | BMT8 | Beta-mannosyltransferase 8 (EC 2.4.1.-) (WRY family protein 3) | 464272 | 0 | Inhibited |
| CAALFM_C306910CA | Q5ADN1 | TPD3 | Protein phosphatase 2A structural subunit | 443227 | 0 | Inhibited |
| CAALFM_C305230WA | P0CY29 | SAP3 | Candidapepsin-3 (EC 3.4.23.24) (ACP 3) (Aspartate protease 3) (Secreted aspartic protease 3) | 437647 | 0 | Inhibited |
| CAALFM_CR01650WA | A0A1D8PS11 | URA5 | Orotate phosphoribosyltransferase (EC 2.4.2.10) | 431889 | 0 | Inhibited |
| CAALFM_CR08210CA | P82611 | ACO1 | Aconitate hydratase, mitochondrial (Aconitase) (EC 4.2.1.3) (Citrate hydro-lyase) | 428531 | 0 | Inhibited |
| CAALFM_C209970CA | Q59YH1 | U1355 | Glucosamine_iso domain-containing protein | 415659 | 0 | Inhibited |
| CAALFM_C304060CA | Q59MR4 | HEM13 | Coproporphyrinogen oxidase (EC 1.3.3.3) | 412379 | 0 | Inhibited |
| CAALFM_C206810CA | A0A1D8PHW1 | RPL11 | Ribosomal 60S subunit protein L11B | 410139 | 0 | Inhibited |
| CAALFM_C106100CA | Q96VB9 | MSI3 | Heat shock protein homolog SSE1 (Chaperone protein MSI3) | 406457 | 0 | Inhibited |
| CAALFM_C305810CA | A0A1D8PKE1 | SKN1 | Skn1p | 405217 | 0 | Inhibited |
| CAALFM_C107690CA | A0A1D8PE78 | U1229 | Importin-alpha export receptor | 394760 | 0 | Inhibited |
| CAALFM_C302280CA | P53704 | GFA1 | Glutamine--fructose-6-phosphate aminotransferase [isomerizing] (GFAT) (EC 2.6.1.16) (D-fructose-6-phosphate amidotransferase) (Hexosephosphate aminotransferase) | 380571 | 0 | Inhibited |
| CAALFM_C110490WA | Q59NB8 | LKH1 | Leucine aminopeptidase 2 (EC 3.4.11.-) (Epoxide hydrolase) (EC 3.3.2.10) (Leukotriene A-4 hydrolase homolog) (LTA-4 hydrolase) | 374407 | 0 | Inhibited |
| CAALFM_C110550CA | A0A1D8PEW1 | GCA2 | Gca2p | 368607 | 0 | Inhibited |
| CAALFM_CR01350CA | Q5A9E7 | U2518 | TPR_REGION domain-containing protein | 363893 | 0 | Inhibited |
| CAALFM_C105670WA | Q5AA00 | U2484 | Peptide hydrolase (EC 3.4.-.-) | 355227 | 0 | Inhibited |
| CAALFM_C103130CA | A0A1D8PCX6 | GGA2 | Phosphatidylinositol 4-phosphate-binding protein | 355042 | 0 | Inhibited |
| CAALFM_C101370CA | A0A1D8PCG7 | RPS21B | 40S ribosomal protein S21 | 353212 | 0 | Inhibited |
| CAALFM_C101910WA | A0A1D8PCL4 | U4532 | Uncharacterized protein | 340898 | 0 | Inhibited |
| CAALFM_CR00580WA | A0A1D8PRQ3 | YRB1 | Ran GTPase-binding protein | 340449 | 0 | Inhibited |
| CAALFM_CR05080WA | A0A1D8PSX0 | VMA13 | V-type proton ATPase subunit H | 333719 | 0 | Inhibited |
| CAALFM_C604280WA | A0A1D8PQD4 | U1085 | E2 ubiquitin-conjugating enzyme (EC 2.3.2.23) | 330738 | 0 | Inhibited |
| CAALFM_CR04660CA | Q59KI0 | UGP1 | UTP--glucose-1-phosphate uridylyltransferase (EC 2.7.7.9) (UDP-glucose pyrophosphorylase) (UDPGP) (UGPase) | 316402 | 0 | Inhibited |
| CAALFM_C503870CA | Q59N81 | U1107 | Uncharacterized protein | 312938 | 0 | Inhibited |
| CAALFM_C101420CA | Q5A904 | CRL1 | Rho family GTPase | 308460 | 0 | Inhibited |
| CAALFM_C404170CA | A0A1D8PM01 | AXL2 | Axl2p | 301149 | 0 | Inhibited |
| CAALFM_C101530CA | A0A1D8PCI5 | U3341 | Arginyl-tRNA synthetase (EC 6.1.1.19) | 298248 | 0 | Inhibited |
| CAALFM_C602860WA | A0A1D8PQ06 | STE23 | Metalloendopeptidase | 293139 | 0 | Inhibited |
| CAALFM_C400070CA | A0A1D8PKX5 | U374 | FXNA-related family protease 1 (Vacuolar membrane protease) | 291774 | 0 | Inhibited |
| CAALFM_C208040CA | A0A1D8PI15 | RPS10 | Ribosomal 40S subunit protein S10A | 281320 | 0 | Inhibited |
| CAALFM_C106450CA | A0A1D8PDT3 | RPS14B | Ribosomal 40S subunit protein S14B | 279711 | 0 | Inhibited |
| CAALFM_C405690WA | Q5A455 | SEC23 | Protein transport protein SEC23 | 274359 | 0 | Inhibited |
| CAALFM_C701800CA | Q5AGZ8 | PFK2 | ATP-dependent 6-phosphofructokinase (ATP-PFK) (Phosphofructokinase) (EC 2.7.1.11) (Phosphohexokinase) | 268155 | 0 | Inhibited |
| CAALFM_CR10490WA | Q9P975 | TIF45 | Eukaryotic translation initiation factor 4E (eIF-4E) (eIF4E) (eIF-4F 25 kDa subunit) (mRNA cap-binding protein) | 263879 | 0 | Inhibited |
| CAALFM_C304500CA | A0A1D8PK40 | RPL19A | Ribosomal protein L19 | 252492 | 0 | Inhibited |
| CAALFM_CR04190WA | Q5A6P9 | SRV2 | Adenylyl cyclase-associated protein | 244649 | 0 | Inhibited |
| CAALFM_C100770CA | Q5ABP8 | ROT1 | Protein ROT1 | 242059 | 0 | Inhibited |
| CAALFM_C104680WA | A0A1D8PDD1 | PIL1 | Lipid-binding protein | 239890 | 0 | Inhibited |
| CAALFM_C500150CA | A0A1D8PMU8 | U5682 | Importin subunit alpha | 239540 | 0 | Inhibited |
| CAALFM_C207810WA | Q5A2K5 | U2200 | L-type lectin-like domain-containing protein | 234138 | 0 | Inhibited |
| CAALFM_C504090CA | Q59LQ6 | SUI1 | Translation initiation factor eIF1 | 233745 | 0 | Inhibited |
| CAALFM_C600760WA | Q59NN8 | FES1 | Hsp70 nucleotide exchange factor FES1 | 231783 | 0 | Inhibited |
| CAALFM_C702240WA | A0A1D8PR25 | FMP45 | Fmp45p | 229416 | 0 | Inhibited |
| CAALFM_C208800CA | A0A1D8PI95 | APE3 | Peptide hydrolase (EC 3.4.-.-) | 227706 | 0 | Inhibited |
| CAALFM_C108410CA | A0A1D8PEB0 | SAM4 | S-adenosylmethionine-homocysteine S-methyltransferase | 222046 | 0 | Inhibited |
| CAALFM_C406990WA | A0A1D8PMQ3 | MNN46 | Uncharacterized protein | 220368 | 0 | Inhibited |
| CAALFM_CR09010CA | A0A1D8PTV7 | U7297 | Putative cystathionine beta-lyase | 220038 | 0 | Inhibited |
| CAALFM_C502170CA | Q5AGF6 | GCD11 | Protein-synthesizing GTPase (EC 3.6.5.3) | 217990 | 0 | Inhibited |
| CAALFM_C602260CA | A0A1D8PPV5 | SEC27 | Coatomer subunit beta' | 216167 | 0 | Inhibited |
| CAALFM_C210690WA | A0A1D8PIS4 | TPS3 | Trehalose 6-phosphate synthase/phosphatase complex subunit | 206585 | 0 | Inhibited |
| CAALFM_C105770CA | A0A1D8PDM9 | PRC3 | Carboxypeptidase (EC 3.4.16.-) | 206126 | 0 | Inhibited |
| CAALFM_C104200CA | A0A1D8PD79 | U1054 | Nucleoporin | 202917 | 0 | Inhibited |
| CAALFM_C505230CA | A0A1D8PP59 | U4016 | Ubiquinol--cytochrome-c reductase subunit | 201757 | 0 | Inhibited |
| CAALFM_C102260CA | A0A1D8PCP6 | AGE3 | Age3p | 201282 | 0 | Inhibited |
| CAALFM_C302960CA | A0A1D8PJN6 | KRE5 | Kre5p | 194202 | 0 | Inhibited |
| CAALFM_C107430WA | Q59WE5 | PHO100 | Pho100p | 192828 | 0 | Inhibited |
| CAALFM_C401690CA | A0A1D8PLC6 | HRT2 | Sugar phosphate phosphatase (EC 3.1.3.-) | 191981 | 0 | Inhibited |
| CAALFM_C105110CA | P79023 | ARO4 | Phospho-2-dehydro-3-deoxyheptonate aldolase, tyrosine-inhibited (EC 2.5.1.54) (3-deoxy-D-arabino-heptulosonate 7-phosphate synthase) (DAHP synthase) (Phospho-2-keto-3-deoxyheptonate aldolase) | 190553 | 0 | Inhibited |
| CAALFM_C305550CA | A0A1D8PKA6 | U6990 | Peptidyl-prolyl cis-trans isomerase (PPIase) (EC 5.2.1.8) | 189279 | 0 | Inhibited |
| CAALFM_C602800WA | A0A1D8PPZ9 | U5553 | L-methionine (R)-S-oxide reductase | 187442 | 0 | Inhibited |
| CAALFM_C111860WA | A0A1D8PF90 | U5281 | Uncharacterized protein | 184547 | 0 | Inhibited |
| CAALFM_C302170CA | A0A1D8PJE0 | LAP41 | Metalloaminopeptidase | 183362 | 0 | Inhibited |
| CAALFM_C114100WA | A0A1D8PFT9 | YPT52 | Rab family GTPase | 182111 | 0 | Inhibited |
| CAALFM_C113300CA | A0A1D8PFL7 | RPN1 | 26S proteasome regulatory subunit RPN1 | 179586 | 0 | Inhibited |
| CAALFM_C503490CA | A0A1D8PNP4 | U6658 | Uncharacterized protein | 178305 | 0 | Inhibited |
| CAALFM_C504260WA | Q5AKB1 | RNY1-B | Ribonuclease T2-like 1-B (RNase T2-like B) (EC 4.6.1.19) | 171907 | 0 | Inhibited |
| CAALFM_C502530WA | A0A1D8PNG6 | UAP1 | UDP-N-acetylglucosamine diphosphorylase | 171194 | 0 | Inhibited |
| CAALFM_C206730WA | Q59KV8 | LSP1 | Lipid-binding protein | 169563 | 0 | Inhibited |
| CAALFM_C700620WA | A0A1D8PQL8 | GUS1 | Glutamyl-tRNA synthetase (EC 6.1.1.17) | 169060 | 0 | Inhibited |
| CAALFM_C504440CA | A0A1D8PNY6 | SFC1 | Sfc1p | 163799 | 0 | Inhibited |
| CAALFM_C503820CA | Q59N76 | BUD7 | Exomer complex subunit | 162484 | 0 | Inhibited |
| CAALFM_C202410WA | A0A1D8PGL1 | U1564 | Endoplasmic reticulum transmembrane protein | 161485 | 0 | Inhibited |
| CAALFM_C603250WA | Q5ABT8 | BMT4 | Beta-mannosyltransferase 4 (EC 2.4.1.-) (WRY family protein 1) | 158224 | 0 | Inhibited |
| CAALFM_C209270CA | A0A1D8PIG5 | SGT1 | Co-chaperone | 155281 | 0 | Inhibited |
| CAALFM_CR09330CA | A0A1D8PTZ1 | U7328 | Probable endonuclease LCL3 (Probable endonuclease lcl3) | 152389 | 0 | Inhibited |
| CAALFM_C700020CA | Q5AFE4 | RVS161 | Regulator of cytoskeleton and endocytosis RVS161 | 149491 | 0 | Inhibited |
| CAALFM_C107700CA | Q5A477 | VRG4 | GDP-mannose transporter (GMT) | 147901 | 0 | Inhibited |
| CAALFM_C204190CA | A0A1D8PH55 | UGA1 | 4-aminobutyrate aminotransferase (EC 2.6.1.19) (GABA aminotransferase) (Gamma-amino-N-butyrate transaminase) | 147782 | 0 | Inhibited |
| CAALFM_C504700CA | A0A1D8PP21 | MES1 | Methionyl-tRNA synthetase (EC 6.1.1.10) | 147089 | 0 | Inhibited |
| CAALFM_C104870WA | Q59VH7 | SAP7 | Candidapepsin-7 (EC 3.4.23.24) (ACP 7) (Aspartate protease 7) (Secreted aspartic protease 7) | 144321 | 0 | Inhibited |
| CAALFM_C202030WA | A0A1D8PGI8 | ARO3 | Phospho-2-dehydro-3-deoxyheptonate aldolase (EC 2.5.1.54) | 142861 | 0 | Inhibited |
| CAALFM_C111120CA | Q59WJ7 | U2304 | Uncharacterized protein | 138330 | 0 | Inhibited |
| CAALFM_C110720CA | Q59WF4 | MNN2 | Alpha-1,2-mannosyltransferase MNN2 (EC 2.4.1.-) | 137883 | 0 | Inhibited |
| CAALFM_CR05780WA | Q59PT0 | VMA2 | V-type proton ATPase subunit B (V-ATPase subunit B) (Vacuolar proton pump subunit B) | 137386 | 0 | Inhibited |
| CAALFM_CR06150CA | A0A1D8PT56 | ADE13 | Adenylosuccinate lyase (ASL) (EC 4.3.2.2) (Adenylosuccinase) | 134888 | 0 | Inhibited |
| CAALFM_C113760WA | Q5AKW4 | AGM1 | Phosphoacetylglucosamine mutase (PAGM) (EC 5.4.2.3) (Acetylglucosamine phosphomutase) (N-acetylglucosamine-phosphate mutase) | 132566 | 0 | Inhibited |
| CAALFM_C603410CA | A0A1D8PQ57 | APA2 | Bifunctional AP-4-A phosphorylase/ADP sulfurylase | 132324 | 0 | Inhibited |
| CAALFM_C112360CA | Q5A3V6 | RIB3 | 3,4-dihydroxy-2-butanone 4-phosphate synthase (DHBP synthase) (EC 4.1.99.12) | 127518 | 0 | Inhibited |
| CAALFM_C108110WA | A0A1D8PEA9 | U5085 | TOG domain-containing protein | 126020 | 0 | Inhibited |
| CAALFM_C406500WA | Q5A1D5 | CDC68 | FACT complex subunit SPT16 (CaCDC68) (Cell division control protein 68) (Facilitates chromatin transcription complex subunit SPT16) | 125326 | 0 | Inhibited |
| CAALFM_C401460CA | A0A1D8PLE6 | U4639 | Uncharacterized protein | 124769 | 0 | Inhibited |
| CAALFM_CR00090CA | A0A1D8PRL3 | U7531 | NAD(P)-bd_dom domain-containing protein | 124666 | 0 | Inhibited |
| CAALFM_CR10160WA | A0A1D8PU56 | FAA4 | Long-chain fatty acid-CoA ligase | 123281 | 0 | Inhibited |
| CAALFM_CR04740CA | Q59MZ5 | ADE6 | Formylglycinamide ribonucleotide amidotransferase (EC 6.3.5.3) (Formylglycinamide ribotide amidotransferase) | 122710 | 0 | Inhibited |
| CAALFM_C205590CA | Q59T45 | U6872 | Putative amidotransferase | 121572 | 0 | Inhibited |
| CAALFM_C105720WA | A0A1D8PDL6 | U2478.1 | Ribosomal 60S subunit protein L7A | 119342 | 0 | Inhibited |
| CAALFM_CR04090CA | Q5A6R2 | ADE17 | 5-aminoimidazole-4-carboxamide ribonucleotide formyltransferase (EC 2.1.2.3) (EC 3.5.4.10) (AICAR transformylase) (IMP synthase) (Inosinicase) (Phosphoribosylaminoimidazolecarboxamide formyltransferase) | 118529 | 0 | Inhibited |
| CAALFM_C208100WA | A0A1D8PI22 | U2175 | Pyr_redox_2 domain-containing protein | 116655 | 0 | Inhibited |
| CAALFM_CR10670WA | Q5ACI8 | CPR6 | Peptidyl-prolyl cis-trans isomerase D (PPIase D) (EC 5.2.1.8) (Rotamase D) | 109607 | 0 | Inhibited |
| CAALFM_C206390CA | Q59Q46 | IMH3 | Inosine-5'-monophosphate dehydrogenase (IMP dehydrogenase) (IMPD) (IMPDH) (EC 1.1.1.205) | 107688 | 0 | Inhibited |
| CAALFM_CR01970CA | A0A1D8PS38 | VMA4 | H(+)-transporting V1 sector ATPase subunit E | 106964 | 0 | Inhibited |
| CAALFM_C401200CA | A0A1D8PL85 | AAT22 | Aspartate aminotransferase (EC 2.6.1.1) | 106612 | 0 | Inhibited |
| CAALFM_C201850WA | A0A1D8PGE3 | RAD23 | UV excision repair protein RAD23 | 105902 | 0 | Inhibited |
| CAALFM_C603760CA | O13426 | SHM2 | Serine hydroxymethyltransferase, cytosolic (SHMT) (EC 2.1.2.1) (Glycine hydroxymethyltransferase) (SHMII) (Serine methylase) | 103672 | 0 | Inhibited |
| CAALFM_C405440CA | A0A1D8PMC1 | U1800 | Uncharacterized protein | 103311 | 0 | Inhibited |
| CAALFM_C703390CA | A0A1D8PRB6 | PUP3 | Proteasome core particle subunit beta 3 | 101530 | 0 | Inhibited |
| CAALFM_C206770WA | A0A1D8PHR2 | U2228 | Uncharacterized protein | 97057 | 0 | Inhibited |
| CAALFM_C207070WA | Q59Z38 | U2262 | NADPH:quinone reductase | 96689 | 0 | Inhibited |
| CAALFM_C113690CA | A0A1D8PFS0 | U5006.1 | SNAP receptor | 95935 | 0 | Inhibited |
| CAALFM_C406610CA | Q5A1E8 | SDH12 | Succinate dehydrogenase [ubiquinone] flavoprotein subunit, mitochondrial (EC 1.3.5.1) | 95457 | 0 | Inhibited |
| CAALFM_C201320WA | A0A1D8PGA6 | RPN9 | Proteasome regulatory particle lid subunit | 93503 | 0 | Inhibited |
| CAALFM_CR01760CA | A0A1D8PS16 | FRS1 | Phenylalanyl-tRNA synthetase beta subunit (EC 6.1.1.20) | 92700 | 0 | Inhibited |
| CAALFM_C107230WA | Q59WC5 | PHO15 | 4-nitrophenylphosphatase (PNPPase) (EC 3.1.3.41) | 87710 | 0 | Inhibited |
| CAALFM_C209750WA | A0A1D8PIF8 | LEU42 | Leu42p | 87666 | 0 | Inhibited |
| CAALFM_C203870WA | Q5AHF9 | GNA1 | Glucosamine 6-phosphate N-acetyltransferase (EC 2.3.1.4) | 85064 | 0 | Inhibited |
| CAALFM_C108260CA | A0A1D8PE91 | LTP1 | Acid phosphatase (EC 3.1.3.2) | 85060 | 0 | Inhibited |
| CAALFM_CR05480WA | A0A1D8PT04 | U3518 | Mitochondrial 2-oxodicarboxylate carrier | 85043 | 0 | Inhibited |
| CAALFM_CR08250CA | A0A1D8PTP9 | HSP104 | Chaperone ATPase | 83188 | 0 | Inhibited |
| CAALFM_CR04380CA | Q5A6M6 | SEC26 | Coatomer subunit beta (Beta-coat protein) | 82952 | 0 | Inhibited |
| CAALFM_C304090WA | Q5ANP6 | SBP1 | Sbp1p | 81410 | 0 | Inhibited |
| CAALFM_C502210WA | A0A1D8PND7 | U4228 | Uncharacterized protein | 79980 | 0 | Inhibited |
| CAALFM_C111170WA | A0A1D8PF22 | WBP1 | Dolichyl-diphosphooligosaccharide--protein glycosyltransferase subunit WBP1 (Oligosaccharyl transferase subunit WBP1) | 78785 | 0 | Inhibited |
| CAALFM_C205550WA | A0A1D8PHE5 | U6867 | Cytochrome b5 heme-binding domain-containing protein | 73405 | 0 | Inhibited |
| CAALFM_C406470WA | A0A1D8PMK4 | U2887 | Uncharacterized protein | 72990 | 0 | Inhibited |
| CAALFM_C504570CA | Q5AK79 | URA7 | CTP synthase (EC 6.3.4.2) (UTP--ammonia ligase) | 71935 | 0 | Inhibited |
| CAALFM_C210470CA | Q5A5S7 | ATG27 | Autophagy-related protein 27 | 71042 | 0 | Inhibited |
| CAALFM_C102230WA | Q59VX8 | SEP7 | Septation protein 7 (Seventh homolog of septin 1) | 66416 | 0 | Inhibited |
| CAALFM_C505020CA | Q5AK26 | U3994 | Dolichyl-diphosphooligosaccharide--protein glycotransferase | 64942 | 0 | Inhibited |
| CAALFM_C305560WA | A0A1D8PK85 | PRE3 | Proteasome endopeptidase complex (EC 3.4.25.1) | 63319 | 0 | Inhibited |
| CAALFM_C301890CA | A0A1D8PJA9 | U1658 | Uncharacterized protein | 63131 | 0 | Inhibited |
| CAALFM_C104150CA | Q59VP4 | U1049 | Nudix hydrolase domain-containing protein | 62027 | 0 | Inhibited |
| CAALFM_C405240CA | Q59R99 | VPH1 | V-type proton ATPase subunit a | 60740 | 0 | Inhibited |
| CAALFM_C303520CA | Q5AEB8 | PRE9 | Proteasome core particle subunit alpha 3 | 60248 | 0 | Inhibited |
| CAALFM_C204490WA | A0A1D8PH93 | U4503 | Uncharacterized protein | 60118 | 0 | Inhibited |
| CAALFM_CR01980CA | Q5A967 | CRC1 | Carnitine:acyl carnitine antiporter | 58963 | 0 | Inhibited |
| CAALFM_C206640CA | A0A1D8PHR0 | VAS1 | Valyl-tRNA synthetase (EC 6.1.1.9) | 54978 | 0 | Inhibited |
| CAALFM_CR03170WA | Q59UF7 | DPS1-1 | Aspartyl-tRNA synthetase (EC 6.1.1.12) | 54825 | 0 | Inhibited |
| CAALFM_C300600WA | Q5A7R7 | IFF11 | Cell wall protein IFF11 (Adhesin-like protein IFF11) | 48111 | 0 | Inhibited |
| CAALFM_C110270CA | Q5AP66 | SFH5 | Phosphatidylinositol transfer protein SFH5 (PITP SFH5) | 47509 | 0 | Inhibited |
| CAALFM_C102150WA | A0A1D8PCN0 | GAL10 | Bifunctional UDP-glucose 4-epimerase/aldose 1-epimerase | 45541 | 0 | Inhibited |
| CAALFM_CR09790WA | O93852 | ALO1 | D-arabinono-1,4-lactone oxidase (ALO) (EC 1.1.3.37) (L-galactono-gamma-lactone oxidase) | 41296 | 0 | Inhibited |
| CAALFM_C202320CA | Q5ALM6 | CPR3 | Peptidyl-prolyl cis-trans isomerase (PPIase) (EC 5.2.1.8) | 40997 | 0 | Inhibited |
| CAALFM_CR01690CA | A0A1D8PS12 | CDC60 | Leucyl-tRNA synthetase (EC 6.1.1.4) | 35806 | 0 | Inhibited |
| CAALFM_C210020CA | Q59XS8 | U1780 | Uncharacterized protein | 31192 | 0 | Inhibited |
| CAALFM_C700340CA | A0A1D8PQJ7 | U7086 | Importin N-terminal domain-containing protein | 30858 | 0 | Inhibited |
| CAALFM_C502300CA | A0A1D8PND9 | RET2 | Coatomer subunit delta | 30401 | 0 | Inhibited |
| CAALFM_C103820WA | A0A1D8PD50 | PDR16 | Phosphatidylinositol transporter | 25609 | 0 | Inhibited |
| CAALFM_C305530WA | A0A1D8PK87 | OST1 | Dolichyl-diphosphooligosaccharide--protein glycosyltransferase subunit 1 | 22665 | 0 | Inhibited |
| CAALFM_C110280CA | Q5AP65 | FMP52 | Protein FMP52, mitochondrial | 20420 | 0 | Inhibited |
| CAALFM_C202720WA | A0A1D8PGN9 | U5834 | Importin N-terminal domain-containing protein | 18548 | 0 | Inhibited |
| CAALFM_C504810WA | Q5AK53 | PFK1 | ATP-dependent 6-phosphofructokinase (ATP-PFK) (Phosphofructokinase) (EC 2.7.1.11) (Phosphohexokinase) | 17492 | 0 | Inhibited |
| CAALFM_C201340WA | A0A1D8PGD2 | U1991 | Uncharacterized protein | 16487 | 0 | Inhibited |
| CAALFM_C209800CA | Q59YF6 | U1370 | Uncharacterized protein | 121986864 | 548176 | Downregulated |
| CAALFM_C603160CA | Q5ABU8 | BMT6 | Beta-mannosyltransferase 6 (EC 2.4.1.-) (WRY family protein 2) | 5500545 | 144150 | Downregulated |
| CAALFM_C403520CA | Q59TP1 | RBT1 | Cell wall protein RTB1 (Repressed by TUP1 protein 1) | 59203399 | 1743680 | Downregulated |
| CAALFM_C703600WA | Q59R28 | MNN26 | Alpha-1,2-mannosyltransferase MNN26 (EC 2.4.1.-) | 4424313 | 274659 | Downregulated |
| CAALFM_CR00180CA | Q5AAH2 | CHT1 | Chitinase 1 (EC 3.2.1.14) | 25351497 | 1817511 | Downregulated |
| CAALFM_C102990CA | P29717 | XOG1 | Glucan 1,3-beta-glucosidase (EC 2.4.1.-) (EC 3.2.1.58) (Exo-1,3-beta-glucanase) | 7091186 | 533204 | Downregulated |
| CAALFM_CR09270CA | P31225 | CBP1 | Corticosteroid-binding protein | 3058451 | 236004 | Downregulated |
| CAALFM_C105890WA | A0A1D8PDR3 | U2460 | Uncharacterized protein | 278713619 | 21884184 | Downregulated |
| CAALFM_C306360CA | A0A1D8PKL0 | CYP5 | Peptidyl-prolyl cis-trans isomerase (PPIase) (EC 5.2.1.8) (Fragment) | 10005243 | 795791 | Downregulated |
| CAALFM_C108330CA | O94038 | ADH2 | Alcohol dehydrogenase 2 (EC 1.1.1.1) | 3502852 | 287807 | Downregulated |
| CAALFM_C108230CA | A0A1D8PEB1 | PLB5 | Lysophospholipase (EC 3.1.1.5) | 7369491 | 638530 | Downregulated |
| CAALFM_C405580CA | Q5A470 | U1239 | Uncharacterized protein | 3205160475 | 307069658 | Downregulated |
| CAALFM_C106940CA | Q5AAU5 | ATC1 | Cell wall acid trehalase ATC1 (EC 3.2.1.28) (Alpha,alpha-trehalase) (Alpha,alpha-trehalose glucohydrolase) | 1656738 | 185452 | Downregulated |
| CAALFM_CR03280WA | A0A1D8PSE7 | IFR2 | Ifr2p | 1830979 | 214819 | Downregulated |
| CAALFM_C202760WA | A0A1D8PGU0 | LHS1 | Hsp70 family chaperone | 2631044 | 325658 | Downregulated |
| CAALFM_C114120CA | Q59ZX3 | RBE1 | Repressed by EFG1 protein 1 (PRY family cell wall protein 2) | 10913110 | 1393111 | Downregulated |
| CAALFM_C402410CA | Q5AF44 | AHP1 | Thioredoxin peroxidase | 2898576 | 432802 | Downregulated |
| CAALFM_CR02140WA | A0A1D8PS50 | RSR1 | Ras family GTPase | 1237248 | 192892 | Downregulated |
| CAALFM_C210210CA | Q59XU5 | RAS1 | Ras-like protein 1 (Ras homolog type B) | 9590623 | 1625024 | Downregulated |
| CAALFM_C111700CA | Q59TU5 | MRF1 | Mrf1p | 1553069 | 267743 | Downregulated |
| CAALFM_C301810CA | Q00310 | MNT1 | Glycolipid 2-alpha-mannosyltransferase 1 (EC 2.4.1.-) (Alpha-1,2-mannosyltransferase 1) | 12286718 | 2134478 | Downregulated |
| CAALFM_C106730WA | A0A1D8PDV5 | RAC1 | Rho family GTPase | 1745031 | 305019 | Downregulated |
| CAALFM_C102520WA | Q5AIB2 | SCW4 | Scw4p | 71841488 | 12619377 | Downregulated |
| CAALFM_C306010WA | A0A1D8PKC3 | CAM1 | Translation elongation factor EF1B gamma | 5196693 | 1028983 | Downregulated |
| CAALFM_C406980WA | P87020 | PRA1 | pH-regulated antigen PRA1 (58 kDa fibrinogen-binding mannoprotein) | 949413136 | 191463192 | Downregulated |
| CAALFM_C105630CA | A0A1D8PDL7 | U2489 | Uncharacterized protein | 2274240 | 471171 | Downregulated |
| CAALFM_C107580CA | Q59PV6 | PRY1 | Secreted protein PRY1 (PRY family protein 1) (Pathogenesis-related protein 1) | 5672511 | 1233693 | Downregulated |
| CAALFM_C206960WA | Q59Z50 | SPE3 | Spermidine synthase | 1248454 | 272056 | Downregulated |
| CAALFM_CR07800WA | P0DJ06 | SAP2 | Candidapepsin-2 (EC 3.4.23.24) (ACP 2) (Aspartate protease 2) (Secreted aspartic protease 2) | 3384003 | 767933 | Downregulated |
| CAALFM_C108030WA | Q5A786 | PFY1 | Profilin | 19838630 | 4821030 | Downregulated |
| CAALFM_C207400CA | Q59U59 | APR1 | Proteinase A | 668275 | 163767 | Downregulated |
| CAALFM_C501580CA | P25997 | CEF3 | Elongation factor 3 (EF-3) (EC 3.6.4.-) | 2296178 | 577419 | Downregulated |
| CAALFM_C201360CA | Q5AD78 | DCW1 | Mannan endo-1,6-alpha-mannosidase DCW1 (EC 3.2.1.101) (Defective cell wall 1) (Endo-alpha-1->6-D-mannanase DCW1) (GPI-anchored protein 51) | 817887 | 208383 | Downregulated |
| CAALFM_CR10110WA | P40954 | CHT3 | Chitinase 3 (EC 3.2.1.14) | 8934916 | 2304862 | Downregulated |
| CAALFM_C203100WA | Q5A0M4 | EFT2 | Elongation factor 2 (EF-2) | 38341925 | 10194807 | Downregulated |
| CAALFM_C600850WA | Q59WW7 | U86 | Glutathione peroxidase | 8910927 | 2438320 | Downregulated |
| CAALFM_C302470CA | Q5AEN2 | RPL9B | Ribosomal 60S subunit protein L9B | 936271 | 257411 | Downregulated |
| CAALFM_C103620CA | Q96W54 | RPS22A | 40S ribosomal protein S22-A | 817165 | 231016 | Downregulated |
| CAALFM_C201380WA | A0A1D8PGF0 | PLB4.5 | Lysophospholipase (EC 3.1.1.5) | 55663308 | 15955247 | Downregulated |
| CAALFM_C304950WA | Q5AND9 | ARF2 | Arf family GTPase | 10436580 | 3002508 | Downregulated |
| CAALFM_C109590CA | Q9P4E5 | LIP10 | Lipase 10 (EC 3.1.1.3) | 937607 | 274292 | Downregulated |
| CAALFM_CR09970WA | A0A1D8PU40 | UBC4 | E2 ubiquitin-conjugating protein | 5620041 | 1644211 | Downregulated |
| CAALFM_C110430WA | A0A1D8PEV2 | PHO8 | Alkaline phosphatase (EC 3.1.3.1) | 1138530 | 350410 | Downregulated |
| CAALFM_C101690CA | Q5A8X6 | LSC1 | Succinate--CoA ligase [ADP-forming] subunit alpha, mitochondrial (EC 6.2.1.5) (Succinyl-CoA synthetase subunit alpha) (SCS-alpha) | 865662 | 273077 | Downregulated |
| CAALFM_C112490WA | A0A1D8PFH5 | PEP1 | Type I sorting receptor | 5027663 | 1590379 | Downregulated |
| CAALFM_C307640CA | A0A1D8PKV4 | FUM12 | Fumarate hydratase (EC 4.2.1.2) | 1457854 | 469632 | Downregulated |
| CAALFM_CR07460CA | A0A1D8PTI1 | U6119 | Uncharacterized protein | 2721770 | 877774 | Downregulated |
| CAALFM_C402280WA | A0A1D8PLI2 | IDI1 | Isopentenyl-diphosphate Delta-isomerase (EC 5.3.3.2) | 1029248 | 337095 | Downregulated |
| CAALFM_C109580CA | A0A1D8PER6 | LIP1 | Triacylglycerol lipase (EC 3.1.1.3) | 6545586 | 2163856 | Downregulated |
| CAALFM_C208290CA | Q59KG2 | RGI1 | Respiratory growth induced protein 1 | 1894748 | 637426 | Downregulated |
| CAALFM_C110160WA | Q5AP79 | MIR1 | Mir1p | 3866605 | 1324388 | Downregulated |
| CAALFM_C600480CA | A0A1D8PPE2 | FET31 | Fet31p | 2303451 | 800525 | Downregulated |
| CAALFM_CR10020CA | Q5ACR4 | YOS9 | Protein OS-9 homolog | 9445768 | 3436504 | Downregulated |
| CAALFM_C202970CA | A0A1D8PGT5 | ALD5 | Aldehyde dehydrogenase (NAD(P)(+)) | 8385820 | 3092237 | Downregulated |
| CAALFM_C603700WA | Q5A8T4 | ALS1 | Agglutinin-like protein 1 (Adhesin 1) | 5836733 | 2166873 | Downregulated |
| CAALFM_C100060WA | P0CY34 | TUP1 | Transcriptional repressor TUP1 | 601680 | 233126 | Downregulated |
| CAALFM_CR09240CA | A0A1D8PTY0 | U7322 | Uncharacterized protein | 2580378 | 1014934 | Downregulated |
| CAALFM_C404050CA | Q5A5U4 | RHD3 | Cell wall protein RHD3 (LDG family protein 7) (Predicted GPI-anchored protein 29) (Repressed during hyphae development protein 3) | 652961 | 260325 | Downregulated |
| CAALFM_C109030CA | Q5APK5 | COX5 | Cytochrome c oxidase subunit Va | 535158 | 223719 | Downregulated |
| CAALFM_C201780WA | A0A1D8PGF8 | MSB2 | Msb2p | 38782073 | 16231156 | Downregulated |
| CAALFM_C103680WA | Q5AIR7 | ENG1 | Endo-1,3(4)-beta-glucanase 1 (Endo-1,3-beta-glucanase 1) (Endo-1,4-beta-glucanase 1) (EC 3.2.1.6) (Daughter specific expression protein 4) (Laminarinase) | 12289384 | 5148838 | Downregulated |
| CAALFM_C107600WA | Q59PV8 | ATP7 | ATP synthase subunit d, mitochondrial | 752558 | 316896 | Downregulated |
| CAALFM_C401750CA | Q9URB4 | FBA1 | Fructose-bisphosphate aldolase (FBP aldolase) (FBPA) (EC 4.1.2.13) (37 kDa major allergen) (Fructose-1,6-bisphosphate aldolase) (IgE-binding allergen) | 52260499 | 22405370 | Downregulated |
| CAALFM_C102860CA | Q5AI79 | YKT6 | Palmitoyltransferase | 1543544 | 661924 | Downregulated |
| CAALFM_C101350CA | P87206 | TIF1 | ATP-dependent RNA helicase eIF4A (EC 3.6.4.13) (Eukaryotic initiation factor 4A) (eIF-4A) (Translation initiation factor 1) | 19704956 | 8495022 | Downregulated |
| CAALFM_C114440CA | A0A1D8PFX8 | GDI1 | Rab GDP dissociation inhibitor | 643743 | 280809 | Downregulated |
| CAALFM_CR07070CA | Q59L12 | ALS3 | Agglutinin-like protein 3 (3D9 antigen) (Adhesin 3) | 1803374440 | 790945441 | Downregulated |
| CAALFM_C104100CA | P83781 | POR1 | Mitochondrial outer membrane protein porin (Cytoplasmic antigenic protein 4) | 11255363 | 4955491 | Downregulated |
| CAALFM_CR07590WA | Q59V63 | EMP24 | Emp24p | 963375 | 425090 | Downregulated |
| CAALFM_C203670WA | A0A1D8PGZ9 | SAP99 | Sap99p | 823603 | 370700 | Downregulated |
| CAALFM_C600820WA | Q59NP5 | SUN41 | Secreted beta-glucosidase SUN41 (EC 3.2.1.-) | 822178567 | 372252831 | Downregulated |
| CAALFM_C113450WA | Q5AL03 | HYR1 | Hyphally regulated cell wall protein 1 (Adhesin-like protein HYR1) | 693967395 | 314484887 | Downregulated |
| CAALFM_CR09100CA | A0A1D8PTW9 | U7306 | Pyridoxine 4-dehydrogenase | 975940 | 444763 | Downregulated |
| CAALFM_C604490WA | Q9P8W1 | LIP4 | Lipase 4 (EC 3.1.1.3) | 129626531 | 59332049 | Downregulated |
| CAALFM_C111450CA | A0A1D8PF68 | SAM2 | S-adenosylmethionine synthase (EC 2.5.1.6) | 4950097 | 2280808 | Downregulated |
| CAALFM_C307830WA | A0A1D8PKW2 | FBP1 | Fructose-bisphosphatase (EC 3.1.3.11) | 1282942 | 594482 | Downregulated |
| CAALFM_C109690WA | Q5APD2 | MLS1 | Malate synthase (EC 2.3.3.9) | 1190948 | 576721 | Downregulated |
| CAALFM_CR02180WA | Q59UY6 | PHO113 | Acid phosphatase (EC 3.1.3.2) | 799660 | 394027 | Downregulated |
| CAALFM_C502400WA | Q5AGD1 | U4248 | Acyl-protein thioesterase 1 (EC 3.1.2.-) | 2216905 | 1103227 | Downregulated |
| CAALFM_C208590WA | Q59Y31 | YWP1 | Yeast-form wall Protein 1 (GPI-anchored protein 24) (Thiol-extractable peptide 1) (flocculation protein 1) | 93511980 | 46731491 | Downregulated |
| CAALFM_C105300CA | Q5A2A7 | PRD1 | Metalloendopeptidase | 1681798 | 854871 | Downregulated |
| CAALFM_C112510WA | A0A1D8PFE5 | RAX2 | Rax2p | 7810323 | 4083069 | Downregulated |
| CAALFM_C209060CA | A0A1D8PIB2 | ASN1 | Asparagine synthase (glutamine-hydrolyzing) (EC 6.3.5.4) | 1248256 | 656104 | Downregulated |
| CAALFM_C208130WA | A0A1D8PI24 | ARA1 | D-arabinose 1-dehydrogenase (NAD(P)(+)) | 337031 | 177257 | Downregulated |
| CAALFM_C113080WA | A0A1D8PFJ9 | OP4 | Op4p | 245750564 | 130583929 | Downregulated |
| CAALFM_C604130CA | A0A1D8PQB9 | ALS4 | Agglutinin-like protein 4 (Adhesin 4) | 7179759 | 3834673 | Downregulated |
| CAALFM_C602730WA | A0A1D8PPY8 | SAC6 | Fimbrin | 547030 | 297714 | Downregulated |
| CAALFM_C602120WA | A0A1D8PPS9 | CHC1 | Clathrin heavy chain (Fragment) | 715684 | 391814 | Downregulated |
| CAALFM_C405320WA | P43065 | LYS1 | Saccharopine dehydrogenase [NAD(+), L-lysine-forming] (SDH) (EC 1.5.1.7) (Lysine--2-oxoglutarate reductase) | 1145361 | 629180 | Downregulated |
| CAALFM_C703670WA | Q59R18 | DED81 | Asparaginyl-tRNA synthetase (EC 6.1.1.22) | 819479 | 457035 | Downregulated |
| CAALFM_C209980WA | A0A1D8PIK6 | U1785 | PI31_Prot_C domain-containing protein | 419250 | 237859 | Downregulated |
| CAALFM_C111660WA | A0A1D8PF79 | GAD1 | Glutamate decarboxylase (EC 4.1.1.15) | 591360 | 346172 | Downregulated |
| CAALFM_CR03500WA | A0A1D8PSH3 | CIT1 | Citrate synthase | 1817356 | 1073367 | Downregulated |
| CAALFM_C401010CA | Q5AMF9 | DAG7 | Dag7p | 1076817 | 667808 | Downregulated |
| CAALFM_C103110WA | A0A1D8PCX8 | RPL6 | 60S ribosomal protein L6 | 1712085 | 1062004 | Downregulated |
| CAALFM_C210030CA | Q59XX2 | MP65 | Cell surface mannoprotein MP65 (EC 3.2.1.-) (Mannoprotein of 65 kDa) (Soluble cell wall protein 10) | 346883511 | 217484633 | Downregulated |
| CAALFM_C402250CA | Q5AMT2 | BGL2 | Glucan 1,3-beta-glucosidase BGL2 (EC 3.2.1.58) (Exo-1,3-beta-glucanase) | 287613159 | 180602740 | Downregulated |
| CAALFM_C702300WA | Q5AH56 | YPS7 | Putative aspartic endopeptidase | 8298943 | 5279480 | Not changed |
| CAALFM_C600440CA | A0A1D8PPC9 | FET34 | Ferroxidase | 11801288 | 7517815 | Not changed |
| CAALFM_C108450CA | P0CY33 | CDC42 | Cell division control protein 42 homolog | 1314418 | 837887 | Not changed |
| CAALFM_C104730CA | Q59VG1 | LYS21 | Homocitrate synthase (EC 2.3.3.14) | 773234 | 493334 | Not changed |
| CAALFM_C601720CA | Q5A4M8 | SUR7 | Protein SUR7 | 1526410 | 979048 | Not changed |
| CAALFM_C107900WA | Q5A799 | COI1 | Coi1p | 12866871 | 8370906 | Not changed |
| CAALFM_C600100CA | A0A1D8PPA5 | HET1 | Het1p | 9985383 | 6507702 | Not changed |
| CAALFM_C113940WA | Q5AKU5 | SIM1 | Secreted beta-glucosidase SIM1 (EC 3.2.1.-) | 56669725 | 37006086 | Not changed |
| CAALFM_CR00620CA | A0A1D8PRR5 | ARG1 | Citrulline--aspartate ligase (EC 6.3.4.5) | 1860367 | 1219518 | Not changed |
| CAALFM_C204310WA | A0A1D8PH52 | ERG10 | Acetyl-CoA C-acetyltransferase | 2349584 | 1568314 | Not changed |
| CAALFM_C114060WA | A0A1D8PFV8 | U7214 | 17-beta-hydroxysteroid dehydrogenase-like protein | 2074298 | 1421882 | Not changed |
| CAALFM_CR06340CA | P83780 | PGI1 | Glucose-6-phosphate isomerase (GPI) (EC 5.3.1.9) (Phosphoglucose isomerase) (PGI) (Phosphohexose isomerase) (PHI) | 1483391 | 1038344 | Not changed |
| CAALFM_C206310CA | Q59P43 | GSP1 | GTP-binding nuclear protein | 1895272 | 1354369 | Not changed |
| CAALFM_C703300CA | Q9P8V9 | LIP8 | Lipase 8 (EC 3.1.1.3) | 1571159 | 1123078 | Not changed |
| CAALFM_C600150WA | A0A1D8PPB1 | ARD | Ardp | 451294 | 324405 | Not changed |
| CAALFM_C203350WA | Q5AHA4 | PGA17 | Predicted GPI-anchored protein 17 | 18845443 | 13669623 | Not changed |
| CAALFM_C110100CA | A0A1D8PES3 | NTF2 | Ran GTPase-binding protein | 596639 | 437893 | Not changed |
| CAALFM_C504270CA | P83783 | SAH1 | Adenosylhomocysteinase (AdoHcyase) (EC 3.3.1.1) (S-adenosyl-L-homocysteine hydrolase) | 22151947 | 16354851 | Not changed |
| CAALFM_C406570CA | P83779 | PDC11 | Pyruvate decarboxylase (EC 4.1.1.1) | 12590093 | 9311522 | Not changed |
| CAALFM_C400220CA | A0A1D8PKZ3 | SUB2 | RNA helicase (EC 3.6.4.13) | 307356 | 228003 | Not changed |
| CAALFM_C305000WA | Q5AND4 | RDI1 | Rdi1p | 8175140 | 6095764 | Not changed |
| CAALFM_C602100WA | A0A1D8PPT3 | U3499 | Uncharacterized protein | 5482999 | 4165455 | Not changed |
| CAALFM_C108990CA | A0A1D8PEG3 | KEX2 | Kexin | 5647562 | 4362940 | Not changed |
| CAALFM_C205500WA | Q59ZE0 | ATP4 | ATP synthase subunit 4, mitochondrial | 923137 | 717424 | Not changed |
| CAALFM_C201430WA | Q5ALX8 | APT1 | Adenine phosphoribosyltransferase (APRT) (EC 2.4.2.7) | 744116 | 581013 | Not changed |
| CAALFM_C603030WA | P43094 | SAP5 | Candidapepsin-5 (EC 3.4.23.24) (ACP 5) (Aspartate protease 5) (Secreted aspartic protease 5) | 4928542009 | 3917854205 | Not changed |
| CAALFM_C500590WA | Q5A516 | PET9 | ADP/ATP translocase (ADP,ATP carrier protein) | 16156377 | 12879319 | Not changed |
| CAALFM_C109660WA | A0A1D8PEM3 | DOA1 | Doa1p | 2868364 | 2323822 | Not changed |
| CAALFM_C503350WA | P83782 | QCR2 | Cytochrome b-c1 complex subunit 2, mitochondrial (Complex III subunit 2) (Core protein II) (Cytoplasmic antigenic protein 5) (Ubiquinol-cytochrome-c reductase complex core protein 2) | 525548 | 427943 | Not changed |
| CAALFM_C103500WA | Q5AI00 | YPT1 | Rab family GTPase | 1958480 | 1594948 | Not changed |
| CAALFM_C304590WA | A0A1D8PK11 | U5917.3 | RNA-binding protein | 642014 | 525572 | Not changed |
| CAALFM_C106480CA | A0A1D8PDT9 | U6264.3 | PRA1 family protein | 224405 | 184518 | Not changed |
| CAALFM_C401840CA | A0A1D8PLD7 | U4609 | DLH domain-containing protein | 989978 | 824117 | Not changed |
| CAALFM_C104020CA | Q59QH2 | CSH1 | Csh1p | 5259643 | 4421702 | Not changed |
| CAALFM_C109680WA | Q5APD4 | MTS1 | Sphingolipid C9-methyltransferase (C-9-MT) (EC 2.1.1.317) (Cyclopropane fatty acyl phospholipid synthase homolog 1) (Methyltransferase for sphingolipid 1) | 579239 | 492806 | Not changed |
| CAALFM_C303870CA | Q59SU1 | SAP9 | Candidapepsin-9 (EC 3.4.23.24) (ACP 9) (Aspartate protease 9) (Secreted aspartic protease 9) | 11874286 | 10224158 | Not changed |
| CAALFM_CR09690CA | A0A1D8PU17 | PLB3 | Lysophospholipase (EC 3.1.1.5) | 1964073 | 1774492 | Not changed |
| CAALFM_C203080WA | A0A1D8PGS5 | IDH2 | Isocitrate dehydrogenase [NAD] subunit, mitochondrial | 217985 | 197074 | Not changed |
| CAALFM_CR10250CA | A0A1D8PU61 | FDH3 | S-(hydroxymethyl)glutathione dehydrogenase (EC 1.1.1.284) | 2482181 | 2249739 | Not changed |
| CAALFM_C402900CA | Q5AFA2 | CRH11 | Extracellular glycosidase CRH11 (EC 3.2.-.-) (Congo red hypersensitive protein 11) | 40946156 | 37222192 | Not changed |
| CAALFM_C703360WA | A0A1D8PRC2 | CPY1 | Carboxypeptidase (EC 3.4.16.-) | 748301 | 683661 | Not changed |
| CAALFM_C504110WA | A0A1D8PNW1 | SCW11 | Putative glucan endo-1\3-beta-D-glucosidase | 19789766 | 18113917 | Not changed |
| CAALFM_C703860WA | A0A1D8PRH0 | U7196 | Proteinase B | 617243 | 565651 | Not changed |
| CAALFM_C400130WA | Q59UT4 | RBT5 | GPI-anchored hemophore RBT5 (Repressed by TUP1 protein 5) | 125029390 | 115913202 | Not changed |
| CAALFM_C306500WA | A0A1D8PKJ3 | UBA1 | E1 ubiquitin-activating protein | 1293105 | 1200522 | Not changed |
| CAALFM_C113050WA | A0A1D8PFL9 | RPL14 | Ribosomal 60S subunit protein L14B | 1589227 | 1479311 | Not changed |
| CAALFM_C600750CA | P46273 | PGK1 | Phosphoglycerate kinase (EC 2.7.2.3) | 37577029 | 34980465 | Not changed |
| CAALFM_C201440CA | A0A1D8PGE0 | U1448.1 | Dynein light chain | 1884280 | 1775862 | Not changed |
| CAALFM_C505390CA | Q5AJY5 | PGA4 | 1,3-beta-glucanosyltransferase PGA4 (EC 2.4.1.-) (GPI-anchored protein 4) | 23652028 | 22292334 | Not changed |
| CAALFM_CR06700CA | Q59PZ6 | SOL3 | 6-phosphogluconolactonase-like protein | 792000 | 749152 | Not changed |
| CAALFM_C301550CA | A0A1D8PJA8 | TOS1 | Tos1p | 28684061 | 27136397 | Not changed |
| CAALFM_C113860CA | A0A1D8PFS4 | GND1 | 6-phosphogluconate dehydrogenase, decarboxylating (EC 1.1.1.44) | 25573260 | 24250645 | Not changed |
| CAALFM_C205030CA | A0A1D8PH96 | RHO3 | Rho family GTPase | 1866572 | 1803799 | Not changed |
| CAALFM_C602330WA | A0A1D8PPV6 | U3475 | Uncharacterized protein | 2977738 | 2879140 | Not changed |
| CAALFM_C210350CA | Q59XW4 | ACS1 | Acetyl-coenzyme A synthetase (EC 6.2.1.1) | 2614676 | 2556247 | Not changed |
| CAALFM_CR02860WA | O42825 | RHO1 | GTP-binding protein RHO1 | 13598968 | 13376729 | Not changed |
| CAALFM_C103030WA | A0A1D8PCW6 | RPS16A | Ribosomal 40S subunit protein S16A | 462192 | 457884 | Not changed |
| CAALFM_C105960WA | Q5AA33 | PGA45 | Predicted GPI-anchored protein 45 | 18938481 | 18963294 | Not changed |
| CAALFM_C700930WA | A0A1D8PQQ3 | GPH1 | Alpha-1,4 glucan phosphorylase (EC 2.4.1.1) | 1112404 | 1114432 | Not changed |
| CAALFM_C501230CA | Q5A1Q0 | U1946 | Glucose-6-phosphate 1-epimerase (EC 5.1.3.15) | 2477791 | 2504917 | Not changed |
| CAALFM_C110290WA | O74254 | GAM1 | Glucoamylase 1 (EC 3.2.1.3) (1,4-alpha-D-glucan glucohydrolase) (Glucan 1,4-alpha-glucosidase) | 5378137 | 5517280 | Not changed |
| CAALFM_C500190CA | Q5A4W7 | FAS1 | Fatty acid synthase subunit beta (EC 2.3.1.86) [Includes: 3-hydroxyacyl-[acyl-carrier-protein] dehydratase (EC 4.2.1.59); Enoyl-[acyl-carrier-protein] reductase [NADH] (EC 1.3.1.9); [Acyl-carrier-protein] acetyltransferase (EC 2.3.1.38); [Acyl-carrier-pr | 4293212 | 4465558 | Not changed |
| CAALFM_CR07790CA | Q59MV9 | YHB1 | Flavohemoprotein (EC 1.14.12.17) (Flavohemoglobin) (Hemoglobin-like protein) (Nitric oxide dioxygenase) (NO oxygenase) (NOD) | 303939 | 317920 | Not changed |
| CAALFM_C501450WA | A0A1D8PN69 | PRC2 | Carboxypeptidase (EC 3.4.16.-) | 881440 | 929873 | Not changed |
| CAALFM_C306590WA | A0A1D8PKJ4 | LYS9 | Saccharopine dehydrogenase (NADP+, L-glutamate-forming) | 1392023 | 1469828 | Not changed |
| CAALFM_C702030WA | P46598 | HSP90 | Heat shock protein 90 homolog | 37115799 | 39592220 | Not changed |
| CAALFM_C107410CA | Q59WE2 | SKP1 | E3 ubiquitin ligase complex SCF subunit | 940281 | 1011909 | Not changed |
| CAALFM_CR10270CA | A0A1D8PU72 | AHA1 | Aha1p | 1049380 | 1139338 | Not changed |
| CAALFM_C210620WA | Q5A5Q8 | RPS4A | 40S ribosomal protein S4 | 546493 | 593775 | Not changed |
| CAALFM_C404530CA | P43076 | PHR1 | pH-responsive protein 1 (pH-regulated protein 1) | 240566734 | 262585747 | Not changed |
| CAALFM_CR01750CA | P0CY31 | SEC4 | Ras-related protein SEC4 | 1054437 | 1151083 | Not changed |
| CAALFM_C701250WA | P83774 | ASC1 | Guanine nucleotide-binding protein subunit beta-like protein (Cytoplasmic antigenic protein 1) | 14796081 | 16270949 | Not changed |
| CAALFM_CR06760CA | A0A1D8PTB5 | LSC2 | Succinate--CoA ligase [ADP-forming] subunit beta, mitochondrial (EC 6.2.1.5) (Succinyl-CoA synthetase beta chain) (SCS-beta) | 544646 | 602706 | Not changed |
| CAALFM_CR08090WA | Q5A397 | SSB1 | Hsp70 family ATPase | 3862187 | 4278624 | Not changed |
| CAALFM_CR00520CA | A0A1D8PRR9 | CRM1 | Exportin | 387494 | 430288 | Not changed |
| CAALFM_C102430CA | A0A1D8PCQ3 | U2930 | Eukaryotic translation initiation factor 2A (eIF-2A) | 174490 | 195005 | Not changed |
| CAALFM_C505050WA | A0A1D8PP43 | ADH1 | Adh1p | 14108199 | 15846929 | Not changed |
| CAALFM_C307440WA | Q9P940 | TPI1 | Triosephosphate isomerase (TIM) (EC 5.3.1.1) (Triose-phosphate isomerase) | 44673301 | 50399771 | Not changed |
| CAALFM_C404470WA | Q5A651 | SAP10 | Candidapepsin-10 (EC 3.4.23.24) (ACP 10) (Aspartate protease 10) (Secreted aspartic protease 10) | 3000162 | 3397583 | Not changed |
| CAALFM_CR07520CA | A0A1D8PTI2 | YPT31 | Rab family GTPase | 700405 | 795600 | Not changed |
| CAALFM_C302110WA | Q5AJF7 | RPL12 | Ribosomal 60S subunit protein L12A | 1342621 | 1526809 | Not changed |
| CAALFM_CR09120CA | A0A1D8PTV4 | TUB1 | Tubulin alpha chain | 1661732 | 1895634 | Not changed |
| CAALFM_C108380WA | Q59QD6 | TEF2 | Elongation factor 1-alpha 2 (EF-1-alpha 2) | 36190067 | 43421933 | Not changed |
| CAALFM_C201120WA | A0A1D8PG96 | KAR2 | Endoplasmic reticulum chaperone BiP (EC 3.6.4.10) (Immunoglobulin heavy chain-binding protein homolog) | 16225488 | 19646609 | Not changed |
| CAALFM_C206870CA | A0A1D8PHR5 | PST1 | Pst1p | 1216890 | 1488350 | Not changed |
| CAALFM_C307150CA | Q5ADQ6 | RPS12 | 40S ribosomal protein S12 | 2610323 | 3204176 | Not changed |
| CAALFM_CR09580CA | A0A1D8PU09 | U6604 | Uncharacterized protein | 245849 | 302041 | Not changed |
| CAALFM_C100400WA | Q5ABA2 | SVF1 | Survival factor 1 | 1434045 | 1770053 | Not changed |
| CAALFM_C110170WA | Q5AP78 | U4886 | Uncharacterized protein | 500021 | 620058 | Not changed |
| CAALFM_C203010CA | Q5A0L0 | RNR21 | Ribonucleotide-diphosphate reductase subunit | 312682 | 391171 | Not changed |
| CAALFM_C702280WA | Q5AH54 | U6484 | Uncharacterized protein | 446552 | 584328 | Not changed |
| CAALFM_C102630CA | Q5AIA1 | EXG2 | Glucan 1,3-beta-glucosidase 2 (EC 3.2.1.58) (Exo-1,3-beta-glucanase 2) | 6700594 | 8789650 | Not changed |
| CAALFM_C200100CA | Q59L72 | PGA52 | GPI-anchored protein 52 | 1196594 | 1578430 | Not changed |
| CAALFM_C503930CA | A0A1D8PNT7 | U3219 | Metallophos domain-containing protein | 988806 | 1328715 | Not changed |
| CAALFM_C102480WA | P31353 | PMM1 | Phosphomannomutase (PMM) (EC 5.4.2.8) | 2363565 | 3198110 | Not changed |
| CAALFM_CR05390WA | A0A1D8PT02 | PST3 | Flavodoxin-like fold family protein | 2176211 | 2949762 | Not changed |
| CAALFM_C400200CA | Q59US5 | MET15 | Bifunctional cysteine synthase/O-acetylhomoserine aminocarboxypropyltransferase | 337499 | 458621 | Not changed |
| CAALFM_C502080CA | A0A1D8PNC7 | HSP12 | Heat shock protein | 2395835 | 3335024 | Not changed |
| CAALFM_C103190CA | A0A1D8PCY4 | ECM33 | Ecm33p | 123090742 | 171507904 | Not changed |
| CAALFM_C504130CA | P40953 | CHT2 | Chitinase 2 (EC 3.2.1.14) | 190967848 | 272488226 | Not changed |
| CAALFM_C602040WA | Q59M70 | MCR1 | NADH-cytochrome b5 reductase 2 (EC 1.6.2.2) (Mitochondrial cytochrome b reductase) | 884086 | 1263398 | Not changed |
| CAALFM_C406540WA | A0A1D8PMM6 | MNN4 | Mnn4p | 449451 | 645603 | Not changed |
| CAALFM_C401520CA | A0A1D8PLC9 | RPL20B | 60S ribosomal protein L20 | 321407 | 473398 | Not changed |
| CAALFM_C501690CA | Q59YH4 | CCT7 | T-complex protein 1 subunit eta (TCP-1-eta) (CCT-eta) | 301448 | 452948 | Not changed |
| CAALFM_C400270WA | A0A1D8PKZ9 | ATP2 | ATP synthase subunit beta (EC 7.1.2.2) | 1767050 | 2674533 | Not changed |
| CAALFM_C404800WA | A0A1D8PM53 | U3799 | Reticulon-like protein | 1293052 | 1979646 | Not changed |
| CAALFM_C402340WA | Q5AF37 | U2769 | Inhibitor I9 domain-containing protein | 1035932 | 1604871 | Not changed |
| CAALFM_C107030CA | Q5AB48 | RBT4 | Secreted protein RBT4 (PRY family protein 4) (Repressed by TUP1 protein 4) | 27161029 | 42704975 | Not changed |
| CAALFM_C102590CA | Q5AIA6 | SNZ1 | Pyridoxal 5'-phosphate synthase (glutamine hydrolyzing) (EC 4.3.3.6) | 1027310 | 1616286 | Not changed |
| CAALFM_C307950CA | O93827 | MPG1 | Mannose-1-phosphate guanyltransferase (EC 2.7.7.13) (ATP-mannose-1-phosphate guanylyltransferase) (CASRB1) (GDP-mannose pyrophosphorylase) | 492465 | 792478 | Not changed |
| CAALFM_C203820CA | A0A1D8PGY8 | RPS9B | Ribosomal 40S subunit protein S9B | 500226 | 848465 | Not changed |
| CAALFM_C602240CA | Q9UVJ4 | RPL10A | 60S ribosomal protein L10a | 11327194 | 19640597 | Not changed |
| CAALFM_C400160CA | A0A1D8PL14 | CAR2 | Ornithine aminotransferase (EC 2.6.1.13) | 7162005 | 12557910 | Not changed |
| CAALFM_C504490CA | A0A1D8PP00 | CAR1 | Arginase (EC 3.5.3.1) | 1120703 | 1983347 | Not changed |
| CAALFM_C703080WA | A0A1D8PR98 | LMO1 | Lmo1p | 208782 | 372508 | Not changed |
| CAALFM_C300640WA | Q5A7S3 | U5393 | Uncharacterized protein | 479050 | 861415 | Not changed |
| CAALFM_C307410CA | Q5ADU2 | KRS1 | Lysine--tRNA ligase (EC 6.1.1.6) (Lysyl-tRNA synthetase) | 173694 | 314738 | Not changed |
| CAALFM_C701640WA | Q5AGX8 | LAT1 | Acetyltransferase component of pyruvate dehydrogenase complex (EC 2.3.1.12) | 395011 | 717523 | Not changed |
| CAALFM_C406920CA | Q5A0X8 | CSA2 | Secreted hemophore CSA2 (Surface antigen protein 2) | 25125963 | 45643371 | Not changed |
| CAALFM_C207140WA | A0A1D8PHU6 | U2269 | Putative phosphoric monoester hydrolase | 177296 | 323809 | Not changed |
| CAALFM_C404390WA | A0A1D8PM41 | U1409.1 | Ribosomal 60S subunit protein L22B | 390877 | 720471 | Not changed |
| CAALFM_C301730CA | Q5AJC0 | UTR2 | Extracellular glycosidase UTR2 (EC 3.2.-.-) (Cell-surface factor 4) | 7488740 | 13866291 | Not changed |
| CAALFM_CR07710WA | A0A1D8PTK1 | ARO2 | Chorismate synthase (EC 4.2.3.5) | 577680 | 1070402 | Not changed |
| CAALFM_C200680CA | Q5AD07 | SOD5 | Cell surface Cu-only superoxide dismutase 5 (EC 1.15.1.1) (Predicted GPI-anchored protein 3) | 235722419 | 441654321 | Not changed |
| CAALFM_C304140CA | Q5ANP2 | EGD2 | Nascent polypeptide-associated complex subunit alpha (NAC-alpha) (Alpha-NAC) | 335013 | 654611 | Not changed |
| CAALFM_C205460WA | P46614 | CDC19 | Pyruvate kinase (PK) (EC 2.7.1.40) | 1846100 | 3659827 | Not changed |
| CAALFM_C700860WA | Q5AFN8 | SSR1 | Covalently-linked cell wall protein 14 | 24207589 | 48538251 | Not changed |
| CAALFM_C502710WA | Q5AG89 | TRR1 | Thioredoxin reductase (EC 1.8.1.9) | 192999 | 388941 | Not changed |
| CAALFM_C307220CA | Q5ADR2 | PRO2 | Glutamate-5-semialdehyde dehydrogenase (EC 1.2.1.41) (Glutamyl-gamma-semialdehyde dehydrogenase) | 426035 | 878903 | Not changed |
| CAALFM_C502890WA | Q5AG68 | YNK1 | Nucleoside diphosphate kinase (EC 2.7.4.6) | 2946639 | 6159502 | Not changed |
| CAALFM_C504300CA | Q5AKA5 | DUG1 | Cys-Gly metallodipeptidase DUG1 (EC 3.4.13.-) (Deficient in utilization of glutathione protein 1) (GSH degradosomal complex subunit DUG1) | 1440067 | 3039654 | Not changed |
| CAALFM_C103220CA | O42766 | BMH1 | 14-3-3 protein homolog | 8300797 | 17838726 | Not changed |
| CAALFM_C601610WA | O94083 | ANB1 | Eukaryotic translation initiation factor 5A (eIF-5A) (eIF-4D) | 1308527 | 2863189 | Not changed |
| CAALFM_C104610WA | A0A1D8PDC4 | ATP1 | ATP synthase subunit alpha | 1309442 | 2881472 | Not changed |
| CAALFM_C503990WA | A0A1D8PNU4 | MID1 | Mid1p | 191786 | 430188 | Not changed |
| CAALFM_CR00200WA | A0A1D8PRM7 | PCK1 | Phosphoenolpyruvate carboxykinase (ATP) (EC 4.1.1.49) | 3104075 | 7306673 | Not changed |
| CAALFM_C209640WA | P34948 | PMI1 | Mannose-6-phosphate isomerase (EC 5.3.1.8) (Phosphohexomutase) (Phosphomannose isomerase) (PMI) | 842394 | 2002070 | Upregulated |
| CAALFM_C700990WA | A0A1D8PQS0 | RPP0 | 60S acidic ribosomal protein P0 | 1039771 | 2494416 | Upregulated |
| CAALFM_C500370WA | A0A1D8PMW6 | COF1 | Actin-depolymerizing factor 1 (Cofilin) | 2537922 | 6117549 | Upregulated |
| CAALFM_C703810WA | A0A1D8PRF5 | SLA2 | Sla2p | 111325 | 273851 | Upregulated |
| CAALFM_C500080CA | A0A1D8PMT6 | U5689 | Coatomer subunit epsilon | 131820 | 325063 | Upregulated |
| CAALFM_C108500CA | P30575 | ENO1 | Enolase 1 (EC 4.2.1.11) (2-phospho-D-glycerate hydro-lyase) (2-phosphoglycerate dehydratase) | 71659864 | 176937709 | Upregulated |
| CAALFM_CR05170CA | Q59QN6 | FDH1 | Formate dehydrogenase (FDH) (EC 1.17.1.9) (NAD-dependent formate dehydrogenase) | 3022358 | 7469907 | Upregulated |
| CAALFM_C305100CA | A0A1D8PK43 | RPL18 | Ribosomal 60S subunit protein L18A | 261838 | 650317 | Upregulated |
| CAALFM_C104240CA | Q59VN4 | HHF1 | Histone H4 | 162738 | 406459 | Upregulated |
| CAALFM_C200660CA | Q5AD05 | SOD4 | Cell surface superoxide dismutase [Cu-Zn] 4 (EC 1.15.1.1) (GPI-anchored protein 2) | 873293 | 2196441 | Upregulated |
| CAALFM_C602710CA | Q5AC08 | SAP6 | Candidapepsin-6 (EC 3.4.23.24) (ACP 6) (Aspartate protease 6) (Secreted aspartic protease 6) | 134230166 | 339580502 | Upregulated |
| CAALFM_C702380CA | P22011 | CYP1 | Peptidyl-prolyl cis-trans isomerase (PPIase) (EC 5.2.1.8) (Cyclophilin) (CPH) (Cyclosporin A-binding protein) (Rotamase) | 18984362 | 48842826 | Upregulated |
| CAALFM_C603850CA | Q5A8I8 | IHD1 | Induced during hyphae development protein 1 (GPI-anchored protein 36) | 9456750 | 24361572 | Upregulated |
| CAALFM_C703250CA | A0A1D8PR99 | PDI1 | Protein disulfide-isomerase (EC 5.3.4.1) | 3046832 | 7896683 | Upregulated |
| CAALFM_CR04480CA | Q5A6L5 | LAP3 | Cysteine proteinase 1, mitochondrial (EC 3.4.22.40) | 579508 | 1502785 | Upregulated |
| CAALFM_C302610CA | Q5AF03 | GLX3 | Glyoxalase 3 (EC 4.2.1.130) (Glutathione-independent glyoxalase) | 12398583 | 32175049 | Upregulated |
| CAALFM_C106930WA | Q5AAU3 | PGA63 | Protein transport protein SEC31 | 477067 | 1243334 | Upregulated |
| CAALFM_C601700WA | A0A1D8PPN6 | RPL32 | Ribosomal 60S subunit protein L32 | 306887 | 807302 | Upregulated |
| CAALFM_C102790WA | Q5AI86 | TIF34 | Eukaryotic translation initiation factor 3 subunit I (eIF3i) (Eukaryotic translation initiation factor 3 39 kDa subunit homolog) (eIF-3 39 kDa subunit homolog) | 62546 | 164664 | Upregulated |
| CAALFM_C101490WA | Q5A8Z9 | U3335 | Uncharacterized protein | 254310 | 686738 | Upregulated |
| CAALFM_C302780WA | Q9HGT6 | SES1 | Serine--tRNA ligase, cytoplasmic (EC 6.1.1.11) (Seryl-tRNA synthetase) (SerRS) (Seryl-tRNA(Ser/Sec) synthetase) | 99101 | 272803 | Upregulated |
| CAALFM_C407180WA | Q5A109 | UBI3 | Ubiquitin-ribosomal 40S subunit protein S31 fusion protein | 12784589 | 35300739 | Upregulated |
| CAALFM_CR05720WA | Q92410 | TPS1 | Alpha,alpha-trehalose-phosphate synthase [UDP-forming] (EC 2.4.1.15) (Trehalose-6-phosphate synthase) (UDP-glucose-glucosephosphate glucosyltransferase) | 349684 | 984162 | Upregulated |
| CAALFM_C304830CA | A0A1D8PK65 | FAS2 | Fatty acid synthase subunit alpha (EC 2.3.1.86) [Includes: 3-oxoacyl-[acyl-carrier-protein] reductase (EC 1.1.1.100); 3-oxoacyl-[acyl-carrier-protein] synthase (EC 2.3.1.41)] | 2126163 | 5990196 | Upregulated |
| CAALFM_C100680WA | Q5ABD3 | ASM3 | Asm3p | 1511102 | 4333333 | Upregulated |
| CAALFM_C604520WA | Q59RI1 | ILS1 | Isoleucyl-tRNA synthetase (EC 6.1.1.5) | 452067 | 1311715 | Upregulated |
| CAALFM_C700750WA | A0A1D8PQN3 | ACB1 | Long-chain fatty acid transporter | 197304 | 585188 | Upregulated |
| CAALFM_C502860CA | P83775 | GRP2 | Putative NADPH-dependent methylglyoxal reductase GRP2 (EC 1.1.1.283) (Cytoplasmic antigenic protein 2) | 1114891 | 3381565 | Upregulated |
| CAALFM_C503610WA | A0A1D8PNR7 | HEX1 | Beta-hexosaminidase (EC 3.2.1.52) | 5321768 | 16148658 | Upregulated |
| CAALFM_C500480CA | P46250 | SEC14 | SEC14 cytosolic factor (Phosphatidylinositol/phosphatidylcholine transfer protein) (PI/PC TP) | 1216369 | 3705157 | Upregulated |
| CAALFM_C204580WA | A0A1D8PH78 | ERG20 | Bifunctional (2E,6E)-farnesyl diphosphate synthase/dimethylallyltranstransferase | 277190 | 849398 | Upregulated |
| CAALFM_C113700WA | A0A1D8PFR4 | ACT1 | Actin | 4903429 | 15086524 | Upregulated |
| CAALFM_CR00860CA | Q5A860 | TMA19 | Translationally-controlled tumor protein homolog (TCTP) | 1736443 | 5358423 | Upregulated |
| CAALFM_C113480WA | P41797 | SSA1 | Heat shock protein SSA1 | 8013415 | 25058097 | Upregulated |
| CAALFM_C107890CA | A0A1D8PE67 | ADE5,7 | AIR synthase (EC 6.3.3.1) (EC 6.3.4.13) (Glycinamide ribonucleotide synthetase) (Phosphoribosyl-aminoimidazole synthetase) (Phosphoribosylamine--glycine ligase) (Phosphoribosylformylglycinamidine cyclo-ligase) (Phosphoribosylglycinamide synthetase) | 197035 | 638567 | Upregulated |
| CAALFM_C109460WA | A0A1D8PEL1 | ERG12 | Mevalonate kinase (MK) (EC 2.7.1.36) | 251489 | 819033 | Upregulated |
| CAALFM_C700710WA | A0A1D8PQN0 | RPS28B | Ribosomal 40S subunit protein S28B | 152836 | 498874 | Upregulated |
| CAALFM_C603320WA | A0A1D8PQ54 | U5620 | Uncharacterized protein | 230483 | 763482 | Upregulated |
| CAALFM_C405780CA | A0A1D8PME3 | CFL2 | Cfl2p | 455728 | 1531933 | Upregulated |
| CAALFM_C301630WA | Q5AJB1 | TFP1 | V-type proton ATPase catalytic subunit A (V-ATPase subunit A) (EC 7.1.2.2) | 470714 | 1602081 | Upregulated |
| CAALFM_CR04510WA | P83776 | HXK2 | Hexokinase-2 (EC 2.7.1.1) (Cytoplasmic antigenic protein 3) (Hexokinase PII) (Hexokinase-B) | 6551451 | 22724330 | Upregulated |
| CAALFM_C109670CA | Q5APD5 | U4830 | Uncharacterized protein | 443593 | 1583419 | Upregulated |
| CAALFM_C306870WA | Q5ADM7 | TDH3 | Glyceraldehyde-3-phosphate dehydrogenase (EC 1.2.1.12) | 14199842 | 51636777 | Upregulated |
| CAALFM_C401900CA | Q5AMP4 | MDH1-1 | Malate dehydrogenase (EC 1.1.1.37) | 1145171 | 4318868 | Upregulated |
| CAALFM_C200340CA | A0A1D8PG20 | ARO8 | Bifunctional 2-aminoadipate transaminase/aromatic-amino-acid:2-oxoglutarate transaminase | 129271 | 489015 | Upregulated |
| CAALFM_C306180CA | P0CU34 | TSA1 | Peroxiredoxin TSA1-A (EC 1.11.1.24) (Thiol-specific antioxidant protein) (Thioredoxin peroxidase) (Thioredoxin-dependent peroxiredoxin TSA1-A) | 12687618 | 48032250 | Upregulated |
| CAALFM_C206920CA | Q59Z54 | U2246 | Smr domain-containing protein | 136172 | 522727 | Upregulated |
| CAALFM_C306250WA | Q5A302 | ERV25 | Endoplasmic reticulum vesicle protein 25 | 6085546 | 23684750 | Upregulated |
| CAALFM_C700110WA | A0A1D8PQH5 | SOD3 | Superoxide dismutase (EC 1.15.1.1) | 1084674 | 4264036 | Upregulated |
| CAALFM_C203270WA | P82612 | GPM1 | Phosphoglycerate mutase (PGAM) (EC 5.4.2.11) (BPG-dependent PGAM) (MPGM) (Phosphoglyceromutase) | 550824 | 2214282 | Upregulated |
| CAALFM_C601070CA | A0A1D8PPI6 | CIP1 | Cip1p | 1113268 | 4526495 | Upregulated |
| CAALFM_C103090WA | P40910 | RPS1 | 40S ribosomal protein S1 (S3aE) | 854094 | 3479422 | Upregulated |
| CAALFM_CR07010WA | Q59SM8 | MIS11 | Formyltetrahydrofolate synthetase (EC 1.5.1.5) (EC 3.5.4.9) (EC 6.3.4.3) (Methenyltetrahydrofolate cyclohydrolase) (Methylenetetrahydrofolate dehydrogenase) | 282501 | 1156614 | Upregulated |
| CAALFM_C304920CA | Q5ANE2 | U5961 | ANK_REP_REGION domain-containing protein | 248569 | 1032580 | Upregulated |
| CAALFM_C100490CA | Q5ABB1 | TTR1 | Dithiol glutaredoxin | 258247 | 1075925 | Upregulated |
| CAALFM_C303170WA | Q5AEF2 | SEC13 | Protein transport protein SEC13 | 171873 | 722422 | Upregulated |
| CAALFM_C406970CA | A0A1D8PMR6 | ZRT101 | Zrt1p | 132684 | 572090 | Upregulated |
| CAALFM_C402360WA | Q5AF38 | AMS1 | Alpha-mannosidase | 13486263 | 58377033 | Upregulated |
| CAALFM_CR03720WA | Q5A017 | TAL1 | Transaldolase (EC 2.2.1.2) | 68233291 | 303990960 | Upregulated |
| CAALFM_C108320WA | Q5A750 | TKL1 | Transketolase (EC 2.2.1.1) | 1350521 | 6137901 | Upregulated |
| CAALFM_C100220WA | O13318 | PHR2 | pH-responsive protein 2 (pH-regulated protein 2) | 854334 | 4011668 | Upregulated |
| CAALFM_CR04810WA | A0A1D8PSV5 | RPS3 | Ribosomal 40S subunit protein S3 | 544975 | 2567656 | Upregulated |
| CAALFM_CR02820WA | A0A1D8PSA9 | PGM2 | Phosphoglucomutase | 3679355 | 18580978 | Upregulated |
| CAALFM_CR00640WA | A0A1D8PRR7 | ACC1 | Biotin carboxylase (EC 6.3.4.14) (EC 6.4.1.2) | 205441 | 1056747 | Upregulated |
| CAALFM_C600870CA | A0A1D8PPG7 | ILV5 | Ketol-acid reductoisomerase, mitochondrial (EC 1.1.1.86) (Acetohydroxy-acid reductoisomerase) (Alpha-keto-beta-hydroxylacyl reductoisomerase) | 225075 | 1180217 | Upregulated |
| CAALFM_C203810CA | A0A1D8PGY0 | RPL21A | Ribosomal 60S subunit protein L21A | 170374 | 915265 | Upregulated |
| CAALFM_C304180WA | Q5ANN9 | KRE9 | Kre9p | 266983 | 1448011 | Upregulated |
| CAALFM_C102110CA | Q59VZ0 | HGT2 | Hgt2p | 245645 | 1365657 | Upregulated |
| CAALFM_C106370CA | Q5AAN7 | PBR1 | Pbr1p | 10296376 | 58404040 | Upregulated |
| CAALFM_C209430WA | Q59LS1 | RPL3 | Ribosomal 60S subunit protein L3 | 158282 | 928500 | Upregulated |
| CAALFM_CR01620CA | P82610 | MET6 | 5-methyltetrahydropteroyltriglutamate--homocysteine methyltransferase (EC 2.1.1.14) (Cobalamin-independent methionine synthase) (Methionine synthase, vitamin-B12 independent isozyme) | 498514 | 2973110 | Upregulated |
| CAALFM_C207380WA | P83784 | SSC1 | Heat shock protein SSC1, mitochondrial (Cytoplasmic antigenic protein 6) (mtHSP70) | 525643 | 3147418 | Upregulated |
| CAALFM_C700960WA | A0A1D8PQQ5 | RPS18 | Ribosomal 40S subunit protein S18B | 1087958 | 6569415 | Upregulated |
| CAALFM_C402070WA | Q5AMR2 | PHO114 | Acid phosphatase (EC 3.1.3.2) | 2215918 | 13933617 | Upregulated |
| CAALFM_CR00540CA | P83778 | MDH1 | Malate dehydrogenase, cytoplasmic (EC 1.1.1.37) | 736467 | 4814139 | Upregulated |
| CAALFM_C104180WA | Q59VP1 | HTB2 | Histone H2B.2 | 400121 | 2640575 | Upregulated |
| CAALFM_C403470CA | Q07730 | ECE1 | Extent of cell elongation protein 1 [Cleaved into: ECE1-I; ECE1-II; Candidalysin ECE1-III; ECE1-IV; ECE1-V; ECE1-VI; ECE1-VII; ECE1-VIII] | 22615411 | 151055914 | Upregulated |
| CAALFM_C703930CA | A0A1D8PRG4 | MAM33 | Mam33p | 115692 | 832270 | Upregulated |
| CAALFM_C502000CA | P83773 | ACH1 | Acetyl-CoA hydrolase (EC 3.1.2.1) (Acetyl-CoA deacylase) (Acetyl-CoA acylase) | 279157 | 2016173 | Upregulated |
| CAALFM_C305220WA | Q5ANA3 | CDR1 | Pleiotropic ABC efflux transporter of multiple drugs CDR1 (Pleiotropic drug resistance protein CDR1) | 195372 | 1418965 | Upregulated |
| CAALFM_C102420CA | A0A1D8PCT0 | GSC1 | 1,3-beta-D-glucan-UDP glucosyltransferase (EC 2.4.1.34) | 546284 | 3968344 | Upregulated |
| CAALFM_C406030WA | A0A1D8PMF8 | CMD1 | Calmodulin | 280379 | 2117816 | Upregulated |
| CAALFM_C500440CA | Q5A501 | VPS2 | ESCRT-III subunit protein | 53963 | 425687 | Upregulated |
| CAALFM_C603720WA | Q5A8K2 | ALA1 | Alanine--tRNA ligase (EC 6.1.1.7) (Alanyl-tRNA synthetase) (AlaRS) | 25366 | 204315 | Upregulated |
| CAALFM_C107120WA | Q59WB3 | GAP4 | S-adenosylmethionine permease GAP4 (SAM permease) (Amino-acid permease GAP4) | 169704 | 1384993 | Upregulated |
| CAALFM_C101060WA | Q5A940 | MBF1 | Multiprotein-bridging factor 1 | 81206 | 671499 | Upregulated |
| CAALFM_CR10350CA | A0A1D8PU69 | TRX1 | Thioredoxin | 6881531 | 65138587 | Upregulated |
| CAALFM_C500930CA | Q5A1M1 | TFS1 | Tfs1p | 54369 | 573811 | Upregulated |
| CAALFM_C205720CA | A0A1D8PHG1 | U6883 | Oxysterol-binding protein | 274176 | 3044190 | Upregulated |
| CAALFM_C300720WA | A0A1D8PJ01 | PMA1 | Plasma membrane ATPase (EC 7.1.2.1) | 1756318 | 19561687 | Upregulated |
| CAALFM_C206680WA | A0A1D8PHP8 | FRP3 | Putative ammonium permease | 142564 | 1685124 | Upregulated |
| CAALFM_C403940CA | A0A1D8PLY4 | PYC2 | Pyruvate carboxylase (EC 6.4.1.1) | 889611 | 10839478 | Upregulated |
| CAALFM_C101300WA | Q5A915 | U3319 | Uncharacterized protein | 765972 | 9683057 | Upregulated |
| CAALFM_C603500CA | Q5A8N2 | SAP4 | Candidapepsin-4 (EC 3.4.23.24) (ACP 4) (Aspartate protease 4) (Secreted aspartic protease 4) | 2999337 | 39602220 | Upregulated |
| CAALFM_C600790CA | Q59NP1 | CTR1 | Copper transport protein CTR1 | 480088 | 7590396 | Upregulated |
| CAALFM_CR02360WA | A0A1D8PS79 | IDP2 | Isocitrate dehydrogenase [NADP] (EC 1.1.1.42) | 476674 | 7561602 | Upregulated |
| CAALFM_C201020WA | Q5AD47 | HGT6 | Hexose transporter | 423582 | 6861940 | Upregulated |
| CAALFM_C501820WA | A0A1D8PN90 | STI1 | Hsp90 cochaperone | 165390 | 3079215 | Upregulated |
| CAALFM_C208810CA | P83777 | IPP1 | Inorganic pyrophosphatase (EC 3.6.1.1) (Pyrophosphate phospho-hydrolase) (PPase) | 2404108 | 47336237 | Upregulated |
| CAALFM_CR10140WA | A0A1D8PU51 | U7590 | Uncharacterized protein | 44791 | 949190 | Upregulated |
| CAALFM_CR07050CA | A0A1D8PTD1 | URA2 | Aspartate carbamoyltransferase (EC 2.1.3.2) (EC 6.3.5.5) | 481280 | 10906490 | Upregulated |
| CAALFM_CR01470WA | Q5A9D4 | CSP37 | Csp37p | 516455 | 12338570 | Upregulated |
| CAALFM_CR08460WA | A0A1D8PTR7 | TPM2 | Tropomyosin | 265677 | 6754016 | Upregulated |
| CAALFM_C104260WA | Q59VN2 | HHT2 | Histone H3.1/H3.2 | 10865 | 292963 | Upregulated |
| CAALFM_C112050WA | Q5A3L0 | RPN2 | 26S proteasome regulatory subunit RPN2 | 201411 | 7387372 | Upregulated |
| CAALFM_C110060CA | A0A1D8PEU0 | U4873 | Uncharacterized protein | 550284 | 36835070 | Upregulated |
| CAALFM_C400120WA | Q59UT5 | PGA7 | GPI-anchored hemophore PGA7 (Predicted GPI-anchored protein 7) (Repressed by TUP1 protein 6) | 8989610 | 936163413 | Upregulated |
